# Genetics of progressive multifocal leukoencephalopathy: update on case reports with an inborn error of immunity and risk variants found in drug-linked cases

**DOI:** 10.3389/fneur.2025.1629581

**Published:** 2025-07-15

**Authors:** Peggy S. Eis, Edward B. Smith, Shapour Jalilzadeh, Eli Hatchwell

**Affiliations:** ^1^Population Bio, Inc., New York, NY, United States; ^2^Population Bio UK, Inc., Oxfordshire, United Kingdom

**Keywords:** hemophagocytic lymphohistiocytosis, inborn error of immunity, immunodeficiency, JC virus, natalizumab, progressive multifocal leukoencephalopathy, rituximab, PML

## Abstract

A genetic predisposition to PML is now substantially supported by case reports of patients molecularly diagnosed with an inborn error of immunity (IEI) and progressive multifocal leukoencephalopathy (PML). Over the past 10 years, 4 IEI genes linked to PML has now grown to 26 as of 2025. Of these 26 genes believed to be causal of an IEI and PML, 24 (92%) are also linked with hemophagocytic lymphohistiocytosis/macrophage activation syndrome (HLH/MAS)—a severe hyper-inflammation syndrome associated with several IEI genes, most notably in 4 genes (*PRF1*, *STX11*, *STXBP2*, *UNC13D*) causing familial forms of the syndrome. Many HLH-linked genes are associated with life-threatening Epstein–Barr virus infections, which analogously suggests JC virus infection plus presence of a pathogenic variant in an HLH-linked IEI gene also increases risk of PML. PML also occurs as a serious adverse event for a subset of immunosuppressive therapies (e.g., natalizumab and rituximab) used to treat patients with immune disorders (e.g., multiple sclerosis and hematological malignancies). Recently, 4 PML risk variants were reported for use in a PML risk test to screen patients who are considering treatment with PML-linked therapies. Interestingly, of the 4 genes with a PML risk variant, 2 (*LY9* and *STXBP2*) cause or are linked to HLH. The aim of our review is two-fold: (1) raise awareness among researchers and clinicians (e.g., neurologists, oncologists, and rheumatologists) that patient genetics are a key risk factor for PML, and (2) further reinforce the rationale for screening at-risk patients for PML risk variants before prescribing a PML-linked drug.

## Introduction

**Figure 1:**
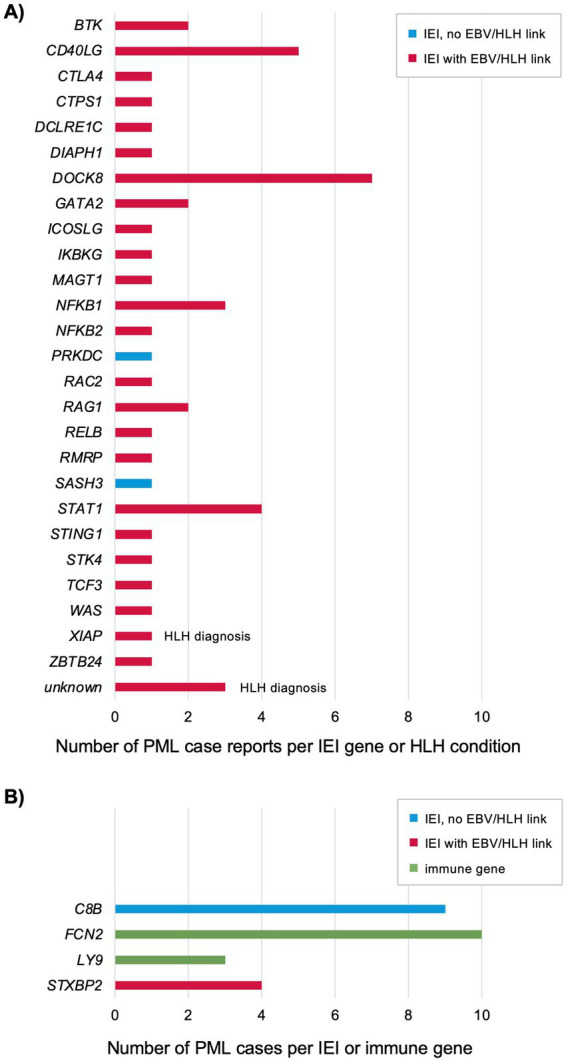
Genes reported for previously published PML cases with an IEI diagnosis, HLH diagnosis, or positive for a PML genetic risk variant (see Supplementary Table 1) (10, 20, 35–38, 96–127). **(A)** Each patient was genetically diagnosed with an IEI plus developed PML or diagnosed with HLH (indicated) and PML. All other PML cases were not reported to also have HLH, but 19 of 26 IEI genes are linked to an increased risk of EBV and/or HLH as associated features (7, 13, 16–28, 30–34, 128). **(B)** Each patient tested positive for a PML risk variant (5, 6). Two genes (*C8B* and *FCN2*) are in the complement pathway (129–133) and two genes (*LY9* and *STXBP2*) are linked to EBV/HLH (see Figure 2).

Progressive multifocal leukoencephalopathy (PML) is a neurological disorder characterized by progressive white matter degeneration. PML occurs as a secondary and often fatal brain disease in immune-suppressed patients infected with human polyomavirus 2 (HPyV2) (1, 2), commonly known as JC virus (JCV) (3, 4). Immune-linked primary diseases associated with an increased risk of PML include HIV infection, hematological malignancies, and autoimmune disorders (3). Treatment of a patient’s primary disease with an immunosuppressant therapy (or non-compliance with antiretroviral therapy in HIV-infected patients) is often a triggering factor for developing PML. We propose that a patient’s underlying genetics are also a key risk factor for developing PML based on two lines of investigation: (1) our genome-wide study of two large cohorts of PML cases revealed four genes that increase PML risk (5, 6) and (2) our assembly of an updated review of the PML case report literature (Figure 1; Supplementary Table 1) on patients diagnosed with an inborn error of immunity (IEI) (7). We note that a majority (73%) of the cases reported in Supplementary Table 1 had a PML diagnosis of definite or probable (3, 8), but diagnostic criteria were not reported for the other PML case reports. PML is an under-appreciated risk in IEI patients and in the wide range of patients on immunosuppressant therapies. Our principal aims are to raise awareness in the clinical communities and increase the vigilance for PML onset, especially in patients with deleterious genetic variants in PML-linked IEI genes.

## Genetic underpinnings of PML—IEI case reports and PML risk variants

Host genetics as an underlying risk factor for PML were first proposed based on a limited number of case reports in patients diagnosed with an IEI and PML (9, 10). The International Union of Immunological Societies (IUIS) Expert Committee has reported there are now 508 IEI genes (7), but none are presently reported to cause an increased risk of PML. In the last 10 years, IEI genes linked to PML based on patients diagnosed with both disorders has increased from 4 to 26 (Figure 1A; Supplementary Table 1). Including PML cases found to have a PML risk variant (Figure 1B; Supplementary Table 1) (6), the total is 28 IEI genes. Notably, a majority of these IEI genes are directly causal or implicated in an increased risk of the hyperinflammation syndrome hemophagocytic lymphohistiocytosis (HLH) (11, 12) and/or severe Epstein–Barr virus (EBV) infections (7, 13), see below for details. Of the four PML risk variant genes (Figure 1B), two are known to cause an IEI, *C8B* is 1 of 33 IEI genes causing complement deficiencies and *STXBP2* is 1 of 7 IEI genes causing familial HLH (FHL) syndromes. Two genes not yet known to cause an IEI are linked to complement and HLH, respectively—*FCN2* is 1 of 3 ficolin genes (*FCN3* is an IEI gene) and *LY9* is linked to the EBV/HLH IEI gene *SH2D1A* via interaction of their protein products—see below for details. We also note that, like IEI in general, there is extensive genetic and phenotypic heterogeneity reported for IEI plus PML genes, with many (21 of 26, 81%) linked to the broader category of common variable immunodeficiency (CVID) (14). Incomplete penetrance is common and attributed to a number of factors, such as digenic/oligogenic/polygenic inheritance (14) and allele-specific expression (termed transcriptotype) (15). Thus, it should not be surprising that many individuals with an IEI are undiagnosed and PML only emerges upon treatment with immunosuppressive drugs (see below and Supplementary Table 2).

## PML and the EBV/HLH connection

Primary HLH (FHL caused by an IEI gene) and secondary HLH (often a complication of rheumatic diseases)—also known as macrophage activation syndrome (MAS) and cytokine storm syndrome (CSS)—are now considered to be a continuum of immune dysfunction (11, 12). The term HLH/MAS has been adopted by experts in the field (12) but, for simplicity, herein will be termed HLH. Since the vast majority of PML case report patients (Figure 1A) were also diagnosed with an IEI linked to EBV/HLH (24 of 26, 92%) (7, 13, 16–34), we also searched the literature and public databases for case reports of patients diagnosed with PML and HLH. We found three cases although genetic information was not reported (unknown genes in Figure 1A; Supplementary Table 1) (35–37). Along with the *XIAP* plus PML case report (38), there are four patients with a diagnosis of PML and HLH. Given the rarity of PML (39, 40) and HLH (11), this is highly unlikely to be a chance association.

PML risk genes *STXBP2* and *LY9* further underscore the connection to EBV/HLH. Familial HLH (FHL syndromes) have a high risk for serious EBV infections (13, 23, 29). Of the four FHL genes, only *STXBP2* (FHL5) is reported to have an inheritance model of autosomal dominant (AD) or autosomal recessive (AR), while all others are AR only (7). All four PML cases with the same *STXBP2* variant were heterozygous (Supplementary Table 1) and in a comparison of natalizumab-treated multiple sclerosis (MS) patients who developed PML (*n* = 2/86) versus matched controls (natalizumab-treated MS patients who did not develop PML, *n* = 0/604) there was a 36-fold increased risk of PML (observed positive predictive value of 100%) (6). While *LY9* is not known to cause an IEI, it is 1 of 9 signaling lymphocytic activation molecule family (SLAMF) members (41, 42) involved in host defense against pathogens (43, 44). SLAMF proteins interact with the protein product of *SH2D1A* (gene alias *SAP*), an IEI gene that causes X-linked lymphoproliferative syndrome (XLP1) characterized by severe EBV infections (13, 23, 29). Another SLAMF gene, *CD48* (gene alias *SLAMF2*), is potentially the first family member linked to an IEI (not yet reported by the IUIS) and HLH based on one case report with a *de novo* variant (45). SLAMF genes are also implicated in cancers (particularly hematological malignancies) (46, 47) and autoimmune diseases such as rheumatoid arthritis (RA) and systemic lupus erythematosus (SLE) (48). Interestingly, the SLAMF locus on chromosome 1 (1q23) harbors all nine SLAMF genes and SLE genetic linkage studies in human (between markers *SPTA1* and *FCGR3A*) (49) and mouse (50) also map to this region. Follow up studies further support the link between SLAMF genes and SLE (51–54).

**Figure 2:**
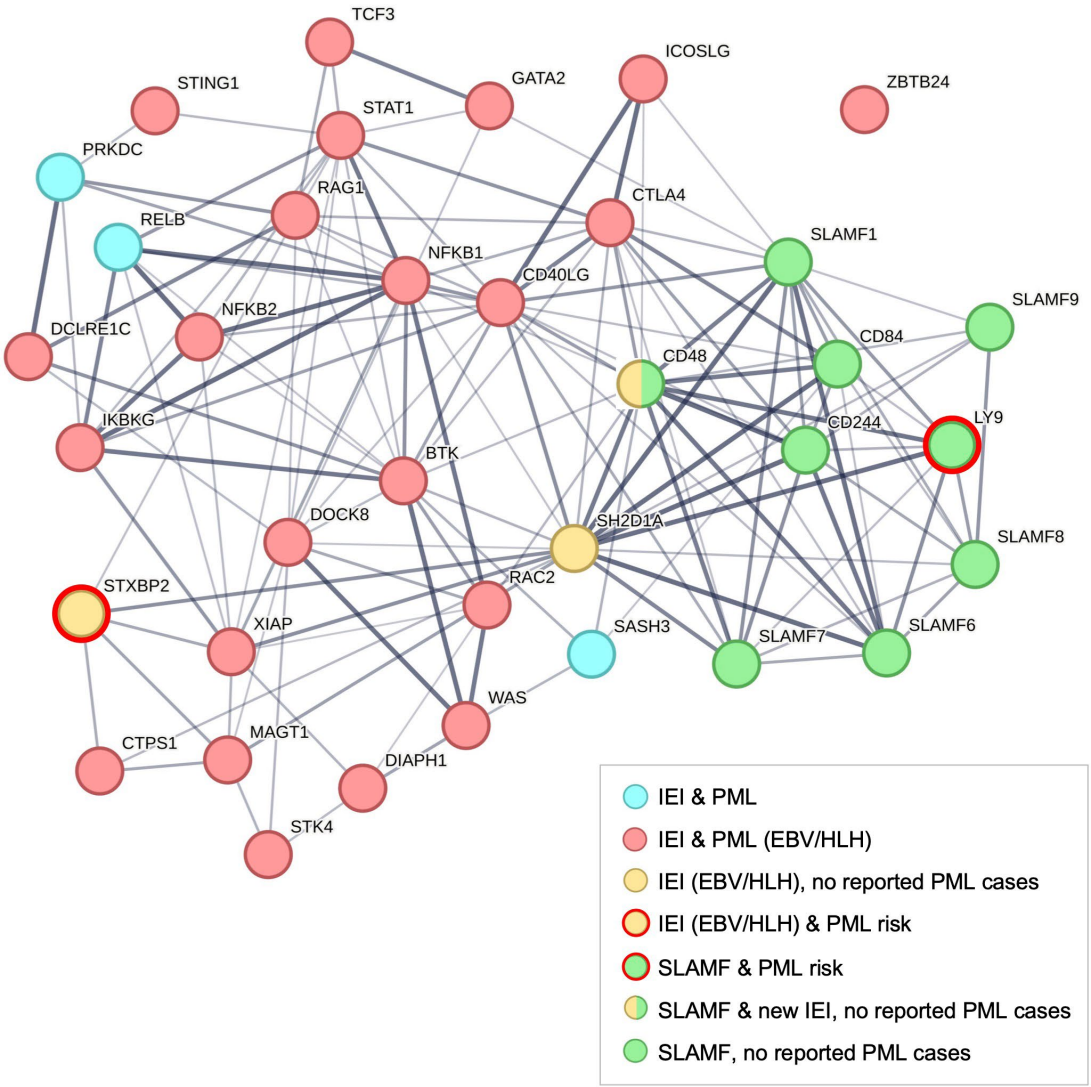
Protein network analysis of PML-linked IEI genes using STRING (55). Default STRING settings were used for 37 genes: 26 IEI + PML case report genes (see Figure 1), 9 SLAMF gene family members (41, 42), SLAMF-interacting gene *SH2D1A* (an IEI gene that causes XLP1) (7), and 2 PML risk genes (SLAMF gene *LY9* and IEI gene *STXBP2*) (5, 6). The protein–protein interaction (PPI) enrichment *p*-value is < 1.0E-16. *ZBTB24* is the only unconnected IEI & PML gene (upper right), see text. SLAMF gene *CD48* (*SLAMF2*) was recently identified as HLH-linked and a potential new IEI, the first member of this gene family found to cause an immune disorder (45, 134). Both IEI and SLAMF genes have been linked to increased risk of autoimmune diseases (48) and hematological cancers (135, 136).

To highlight the interactions between PML-linked IEI and other immune genes, we performed a protein network analysis using STRING (Figure 2) (55). The analysis included 37 genes: 26 IEI plus PML case report genes (Figure 1A), *SH2D1A*, PML risk variant genes (*STXBP2* and *LY9*, but not complement pathway genes *C8B* and *FCN2*), and other SLAMF genes. All genes had multiple connections except *ZBTB24* (Figure 2, upper right). However, 14 of 26 IEI plus PML genes (including *ZBTB24*) are linked to natural killer (NK) cell deficiency or impaired NK cell function (56, 57). PML risk genes *LY9* and *STXBP2* are also linked to NK cell function (57–59). Finally, while several complement system genes cause IEI (including PML risk gene *C8B*) (7), they are not extensively linked to HLH. However, more recent studies do show coexistence of defects in complement and HLH (60), particularly in patients diagnosed with both HLH and thrombotic microangiopathy (TMA) (61, 62).

## Functional evidence for PML-linked genes and JCV

For detailed background on JCV biology, see three recent reviews (4, 63, 64). One of the earliest links between JCV and an IEI gene is a study (65) that found JCV’s agnoprotein interacts with the protein product of *XRCC6* (gene alias *KU70*, a DNA repair protein) and impairs function of the protein product of IEI gene *PRKDC* (66), which causes DNA-PKcs deficiency (7). Subsequent work (67, 68) by this group identified JCV protein links to two other DNA repair genes, IEI genes *DCLRE1C* (gene alias *ARTEMIS*) and *RAD51* (a cause of Fanconi anemia) (7). PML case reports were found for patients with mutations in *DCLRE1C* and *PRKDC* (Figure 1A; Supplementary Table 1). Another group (69) reported an interaction between JCV’s agnoprotein and the protein product of *AP3D1*, which is an IEI gene that causes an FHL syndrome (Hermansky-Pudlak, type 10) (7). The agnoprotein-AP3D1 interaction was validated in a proteomics study (70). Adapter protein (AP) complexes are comprised of host gene proteins, such as AP3D1, that many viruses hijack for viral propagation and evading host immune responses (71).

Further evidence supporting PML-linked genes comes from a recent study (72) using proteomic and single cell RNA sequencing methods on cerebrospinal fluid (CSF) and serum samples from PML patients. Top genes/proteins from these analyses included chemokines and their receptors (e.g., CCL4, CCL5, CCR2, CCR5, CXCR3, CXCR6) as a key feature in PML versus non-PML samples. This is not surprising given their role in NK cell biology (58, 59), but this study also highlighted a link to PML risk genes *LY9* and *STXBP2* (6). We observed the following genes in the top quartile (25%, average log2 fold change) of genes in the RNA sequencing data: CD4-PML cluster vs. other CD4 + T cells included PML risk gene *STXBP2*, 2 SLAMF genes (*SLAMF1*, *SLAMF6*), 8 PML plus IEI case report genes (*CD40LG*, *DOCK8*, *IKBKG*, *RAC2*, *RMRP*, *SASH3*, *STAT1*, *WAS*), and *ITGA4* (target of natalizumab); CD8-PML cluster vs. other CD8 + T cells included *SLAMF7*, 9 PML plus IEI case report genes (*CTPS1*, *DIAPH1*, *DOCK8*, *RAC2*, *RELB*, *RMRP*, *SASH3*, *STAT1*, *WAS*), and *ITGA4*.

Like other IEI disorders, severe infection risk is increased for some genes/viruses, although the IUIS has yet to add risk of PML due to JCV infection to a subset of their current list of 508 IEI genes (7), likely because PML from JCV infection is a rarer entity in IEI patients. We note two key parallels between EBV and JCV causing severe, life-threatening infections in IEI patients and those with milder immunodeficiency (e.g., lymphoma and SLE patients): (1) the ubiquitous presence of these viruses in worldwide populations (EBV > 90%, JCV 60–80%) (4, 13) wherein the infection is usually asymptomatic or relatively benign, and (2) both viruses are linked to HLH, which we think the current evidence suggests is due to host genetics (Figure 1; Supplementary Table 1). While co-infection with HIV and JCV leading to PML was common before the era of antiretroviral therapies (3, 4, 73), not much is known about patients co-infected with EBV and JCV. Two interesting observations that will hopefully lead to further research are EBV-JCV recombination leading to increased neurovirulence of JCV (74) and a case report of a HIV-infected patient who developed primary central nervous system lymphoma with tumors infected with both EBV and JCV (75).

## Iatrogenic (drug-linked) PML as a serious adverse event (SAE) is not going away

In the first two epochs of reported PML cases, hematological malignancies and acquired immunodeficiency syndrome (AIDS) due to HIV infection were the main risk factors (3, 4). Around 2002–2005, reports of iatrogenic PML (i.e., drug-linked) were emerging (3). PML is now recognized as a significant risk factor in a wide range of patients treated with a wide range of immunosuppressive therapies for their primary disease. The highest number of drug-linked PML cases to date are from natalizumab (used to treat MS and Crohn’s disease) and rituximab, which is primarily used to treat cancers and autoimmune disorders (76–78). To assess the current landscape of drugs with the highest PML risk, we used the FDA Adverse Event Reporting System (FAERS)^1^ to identify the number of PML cases reported after treatment with a given drug (Supplementary Table 2). The FAERS database is an excellent resource even though underreporting is a limitation of this database (79–81) and not all reported PML cases will have been validated as definite/probable PML (3, 8). Our FAERS analysis focused on the past 5 years (2020–2024) in order to better represent the current situation for older drugs plus highlight newer drugs with an appreciable number of PML cases. To minimize counting duplicate reports of PML cases, we filtered the data using the original manufacturer (Sender) for a given drug, although this may result in an underreporting of PML cases linked to generic drugs. Also, since natalizumab is the highest risk PML-linked drug, when filtering the data we excluded instances for a given drug if natalizumab was also listed (i.e., oftentimes multiple drugs are listed for a given PML case). Drug data in Supplementary Table 2 are grouped according to four main indications (MS, hematological malignancies, non-MS autoimmune diseases such as RA and SLE, and other). We also highlighted PML risk drugs with a boxed warning (the highest warning issued by the FDA) for PML. Finally, we listed some newer drugs that have yet to report a PML case to the FDA if it had the same mechanism of action (MOA) as drugs already linked to PML.

Even after excluding the large number of historical PML cases (i.e., before 2020), the two highest risk drugs continue to be natalizumab and rituximab. We note rituximab PML cases are listed under 3 of 4 subsections of Supplementary Table 2, reflecting its use to treat a wide range of disorders. For MS drugs, natalizumab (*n* = 231) and fingolimod (*n* = 51, 58 for all drugs targeting S1P modulators) had the highest number of PML cases but ocrelizumab (*n* = 28) and other CD20-targeting drugs also have an appreciable number of reported PML cases (44 total in the MS section for all CD20 drugs). Importantly, we noted FAERS now reports instances of natalizumab patients treated with extended interval dosing (EID, also termed Q6W) (82, 83), which is reported under the Reactions column as “Prescribed Underdose.” About a third of natalizumab FAERS PML cases were classified as “prescribed underdose” but this did not reduce the death rate: regular dose 155 PML cases and 17% died vs. 76 underdose and 21% died.

Interestingly, we note that some MS drugs have been linked to cases of HLH (84–88), although we did not find any case reports of patients treated with these drugs who developed HLH and PML. Importantly, several IEI genes are also drug targets (Supplementary Table 2): *BTK*, *C5*, *CD19*, *CD20* (gene symbol now *MS4A1*), *CD3D*, *CD3E*, *CD3G*, *CD79B*, *JAK1*, and *JAK3* (7). The PML-linked drug belimumab targets *TNFSF13B* (gene aliases *BAFF* and *BLYS*), the ligand of IEI genes *TNFRSF13B* (gene alias *TACI*) and *TNFRSF13C* (gene alias *BAFFR*) (89). We also note there are several case reports of multiple myeloma (MM) patients diagnosed with PML (90–93). Reported drugs for a subset of these cases included bortezomib, daratumumab, ixazomib, lenalidomide, pomalidomide, and thalidomide. All of these MM drugs have been linked to ≥ 3 PML cases in FAERS (Supplementary Table 2). Elotuzumab, an MM drug that targets HLH-linked gene *SLAMF7*, has 5 PML cases reported in FAERS and for one case report the MM patient developed PML during treatment with lenalidomide and elotuzumab (93). There are presently limited or no warnings of PML in the prescribing information for these MM drugs (Supplementary Table 2) despite the growing number of PML cases reported to FAERS (e.g., in the last 5 years, there are 27 PML cases reported for daratumumab but still no warning of PML in its prescribing information). These observations, in concert, underscore the delicate balance of the immune system in having too much or too little of a given IEI gene product.

For clinicians and regulators, the key points to consider are: (1) drug-linked cases of PML occur for a wide range of drugs and primary diseases, (2) efforts to mitigate risk for natalizumab (e.g., EID/Q6W treatment regimen and regular JCV antibody testing) are insufficient, (3) additional early detection measures (more frequent brain MRIs, JCV testing, and other biomarkers) could be implemented for higher risk patients (not just MS patients), and (4) preventive testing for PML risk genetic variants/genes (6) may help reduce the number of drug-linked PML cases (94). Given that PML is often life-threatening, up-to-date information should be made available to clinicians, including via prescribing information (i.e., drug labels) (95), to better inform clinicians about recent advances in genetic testing for PML risk.

## Constellation of PML risk factors: five recommendations for clinicians and regulators

Based on the continuing increase in PML plus IEI case reports and PML cases that carry a PML risk variant (Figure 1; Supplementary Table 1), a predominant risk factor of PML appears to be host genetics (Supplementary Figure 1). Primary diseases (each with their own predisposing genetic variants in immune-linked genes), immunosuppressant drugs, and infections (JCV is required but co-infection with HIV increases the risk and this may be true for other viruses linked to severe infections in IEI patients) also provide multiple pathways leading to the development of PML. Since there are no approved treatments for PML, prevention is the best defense. Therefore, we propose that experts in the field consider the following recommendations for increased vigilance of PML: (1) add JCV and PML to IUIS tables of IEI, (2) consider HLH/MAS (both primary and secondary) to be a concomitant risk factor of PML, (3) use the PML risk genetic test in all at risk patients (i.e., included but not limited to MS patients prior to treatment with natalizumab), (4) for at risk patients, such as carriers of the PML risk variants, implement more frequent brain MRIs plus more frequent and widespread JCV DNA and antibody testing, and (5) promote PML awareness campaigns to patients and clinicians for other diseases (and immunosuppressant drugs used to treat them) besides the MS community.

## Footnotes

^1^ https://www.fda.gov/drugs/surveillance/fdas-adverse-event-reporting-system-faers

## References

[1] Ehlers B Anoh AE Ben Salem N Broll S Couacy-Hymann E Fischer D. Novel polyomaviruses in mammals from multiple orders and reassessment of polyomavirus evolution and taxonomy. Viruses. (2019) 11:930. doi: 10.3390/v11100930, PMID: 31658738 PMC6833039

[2] Polyomaviridae Study Group of the International Committee on Taxonomy of Viruses Calvignac-Spencer S Feltkamp MC Daugherty MD Moens U Ramqvist T. A taxonomy update for the family Polyomaviridae. Arch Virol. (2016) 161:1739–50. doi: 10.1007/s00705-016-2794-y, PMID: 26923930

[3] Cortese I Reich DS Nath A. Progressive multifocal leukoencephalopathy and the spectrum of JC virus-related disease. Nat Rev Neurol. (2021) 17:37–51. doi: 10.1038/s41582-020-00427-y, PMID: 33219338 PMC7678594

[4] Rocchi A Sariyer IK Berger JR. Revisiting JC virus and progressive multifocal leukoencephalopathy. J Neurovirol. (2023) 29:524–37. doi: 10.1007/s13365-023-01164-w, PMID: 37659983

[5] Eis PS Bruno CD Richmond TA Koralnik IJ Hanson BA Major EO. Germline genetic risk variants for progressive multifocal leukoencephalopathy. Front Neurol. (2020) 11:186. doi: 10.3389/fneur.2020.00186, PMID: 32256442 PMC7094807

[6] Hatchwell E Smith EB III Jalilzadeh S Bruno CD Taoufik Y Hendel-Chavez H. Progressive multifocal leukoencephalopathy genetic risk variants for pharmacovigilance of immunosuppressant therapies. Front Neurol. (2022) 13:1016377. doi: 10.3389/fneur.2022.1016377 36588876 PMC9795231

[7] Poli MC Aksentijevich I Bousfiha AA Cunningham-Rundles C Hambleton S Klein C. Human inborn errors of immunity: 2024 update on the classification from the International Union of Immunological Societies Expert Committee. J Hum Immun. (2025) 1:e20250003. doi: 10.70962/jhi.20250003 PMC12829761 41608114

[8] Berger JR Aksamit AJ Clifford DB Davis L Koralnik IJ Sejvar JJ. PML diagnostic criteria: consensus statement from the AAN neuroinfectious disease section. Neurology. (2013) 80:1430–8. doi: 10.1212/WNL.0b013e31828c2fa1, PMID: 23568998 PMC3662270

[9] Hatchwell E. Is there a (host) genetic predisposition to progressive multifocal leukoencephalopathy? Front Immunol. (2015) 6:216. doi: 10.3389/fimmu.2015.00216, PMID: 26029204 PMC4426763

[10] Zerbe CS Marciano BE Katial RK Santos CB Adamo N Hsu AP. Progressive multifocal leukoencephalopathy in primary immune deficiencies: stat 1 gain of function and review of the literature. Clin Infect Dis. (2016) 62:986–94. doi: 10.1093/cid/civ1220, PMID: 26743090 PMC4803104

[11] Henter JI. Hemophagocytic Lymphohistiocytosis. N Engl J Med. (2025) 392:584–98. doi: 10.1056/NEJMra2314005, PMID: 39908433

[12] Shakoory B Geerlinks A Wilejto M Kernan K Hines M Romano M. The 2022 EULAR/ACR points to consider at the early stages of diagnosis and management of suspected haemophagocytic lymphohistiocytosis/macrophage activation syndrome (HLH/MAS). Ann Rheum Dis. (2023) 82:1271–85. doi: 10.1136/ard-2023-224123, PMID: 37487610 PMC11017727

[13] Latour S. Human immune responses to Epstein-Barr virus highlighted by Immunodeficiencies. Annu Rev Immunol. (2025) 43:723–49. doi: 10.1146/annurev-immunol-082323-035455, PMID: 40279309

[14] Peng XP Caballero-Oteyza A Grimbacher B. Common variable immunodeficiency: more pathways than roads to Rome. Annu Rev Pathol. (2023) 18:283–310. doi: 10.1146/annurev-pathmechdis-031521-024229, PMID: 36266261

[15] Stewart O Gruber C Randolph HE Patel R Ramba M Calzoni E. Monoallelic expression can govern penetrance of inborn errors of immunity. Nature. (2025) 637:1186–97. doi: 10.1038/s41586-024-08346-4, PMID: 39743591 PMC11804961

[16] Boast B Goel S Gonzalez-Granado LI Niemela J Stoddard J Edwards ESJ. TCF3 haploinsufficiency defined by immune, clinical, gene-dosage, and murine studies. J Allergy Clin Immunol. (2023) 152:736–47. doi: 10.1016/j.jaci.2023.05.017, PMID: 37277074 PMC10527523

[17] Canna SW Marsh RA. Pediatric hemophagocytic lymphohistiocytosis. Blood. (2020) 135:1332–43. doi: 10.1182/blood.2019000936, PMID: 32107531 PMC8212354

[18] Fan H Yang Z Wu Y Lu X Li T Lu X. Human inborn errors of immunity underlying Talaromyces marneffei infections: a multicenter, retrospective cohort study. Front Immunol. (2025) 16:1492000. doi: 10.3389/fimmu.2025.1492000, PMID: 39911395 PMC11794527

[19] Kamae C Imai K Kato T Okano T Honma K Nakagawa N. Clinical and immunological characterization of ICF syndrome in Japan. J Clin Immunol. (2018) 38:927–37. doi: 10.1007/s10875-018-0559-y, PMID: 30353301

[20] Kaustio M Nayebzadeh N Hinttala R Tapiainen T Astrom P Mamia K. Loss of DIAPH1 causes SCBMS, combined immunodeficiency, and mitochondrial dysfunction. J Allergy Clin Immunol. (2021) 148:599–611. doi: 10.1016/j.jaci.2020.12.656, PMID: 33662367

[21] Klemann C Camacho-Ordonez N Yang L Eskandarian Z Rojas-Restrepo JL Frede N. Clinical and immunological phenotype of patients with primary immunodeficiency due to damaging mutations in NFKB2. Front Immunol. (2019) 10:297. doi: 10.3389/fimmu.2019.00297, PMID: 30941118 PMC6435015

[22] La Rosée P Horne A Hines M von Bahr Greenwood T Machowicz R Berliner N. Recommendations for the management of hemophagocytic lymphohistiocytosis in adults. Blood. (2019) 133:2465–77. doi: 10.1182/blood.2018894618, PMID: 30992265

[23] Latour S Fischer A. Signaling pathways involved in the T-cell-mediated immunity against Epstein-Barr virus: lessons from genetic diseases. Immunol Rev. (2019) 291:174–89. doi: 10.1111/imr.12791, PMID: 31402499

[24] Leopizzi M Mundo L Messina E Campolo F Lazzi S Angeloni A. Epstein-Barr virus-encoded EBNA2 downregulates ICOSL by inducing mi R-24 in B-cell lymphoma. Blood. (2024) 143:429–43. doi: 10.1182/blood.2023021346, PMID: 37847858 PMC10862363

[25] Münz C. Co-stimulatory molecules during immune control of Epstein Barr virus infection. Biomol Ther. (2021) 12:38. doi: 10.3390/biom12010038, PMID: 35053187 PMC8774114

[26] Qiu KY Liao XY Wu RH Huang K Fang JP Zhou DH. X-linked hyper-IgM syndrome: a phenotype of Crohn's disease with Hemophagocytic Lymphohistiocytosis. Pediatr Hematol Oncol. (2017) 34:428–34. doi: 10.1080/08880018.2017.1409301, PMID: 29303623

[27] Sathishkumar D Gach JE Ogboli M Desai M Cole T Hogler W. Cartilage hair hypoplasia with cutaneous lymphomatoid granulomatosis. Clin Exp Dermatol. (2018) 43:713–7. doi: 10.1111/ced.13543, PMID: 29744913

[28] Schulert GS Cron RQ. The genetics of macrophage activation syndrome. Genes Immun. (2020) 21:169–81. doi: 10.1038/s41435-020-0098-4, PMID: 32291394

[29] Tangye SG Latour S. Primary immunodeficiencies reveal the molecular requirements for effective host defense against EBV infection. Blood. (2020) 135:644–55. doi: 10.1182/blood.2019000928, PMID: 31942615

[30] Thomsen MM Skouboe MK Mohlenberg M Zhao J de Keukeleere K Heinz JL. Impaired STING activation due to a variant in the E3 ubiquitin ligase AMFR in a patient with severe VZV infection and Hemophagocytic Lymphohistiocytosis. J Clin Immunol. (2024) 44:56. doi: 10.1007/s10875-024-01653-5, PMID: 38277122 PMC10817851

[31] Yan H Mo Y Liu S Luo X Liu L Zhou L. Case report: Hemophagocytic lymphohistiocytosis in a child with primary immunodeficiency infected with Talaromyces marneffei. Front Immunol. (2022) 13:1038354. doi: 10.3389/fimmu.2022.1038354, PMID: 36532052 PMC9755863

[32] Zeng Q Jin Y Yin G Yang D Li W Shi T. Peripheral immune profile of children with Talaromyces marneffei infections: a retrospective analysis of 21 cases. BMC Infect Dis. (2021) 21:287. doi: 10.1186/s12879-021-05978-z, PMID: 33743629 PMC7980795

[33] Zhang L Lv G Peng Y Yang L Chen J An Y. A novel RAC2 mutation causing combined immunodeficiency. J Clin Immunol. (2023) 43:229–40. doi: 10.1007/s10875-022-01373-8, PMID: 36190591

[34] Zhang W Chen X Gao G Xing S Zhou L Tang X. Clinical relevance of gain- and loss-of-function germline mutations in STAT1: a systematic review. Front Immunol. (2021) 12:654406. doi: 10.3389/fimmu.2021.654406, PMID: 33777053 PMC7991083

[35] Ishikawa Y Kasuya T Ishikawa J Fujiwara M Kita Y. A case of developing progressive multifocal leukoencephalopathy while using rituximab and mycophenolate mofetil in refractory systemic lupus erythematosus. Ther Clin Risk Manag. (2018) 14:1149–53. doi: 10.2147/TCRM.S167109, PMID: 29983569 PMC6027819

[36] Koop A Zaied A Yataco J. A case of progressive multifocal leukoencephalopathy in a patient with EBV-associated hemophagocytic lymphohistiocytosis. Chest. (2016) 150:374A. doi: 10.1016/j.chest.2016.08.38727071810

[37] Kumar A. The newly available FAERS public dashboard: implications for health care professionals. Hosp Pharm. (2019) 54:75–7. doi: 10.1177/0018578718795271, PMID: 30923396 PMC6431724

[38] Seguier J Briantais A Ebbo M Meunier B Aurran T Coze S. Late-onset progressive multifocal leukoencephalopathy (PML) and lymphoma in a 65-year-old patient with XIAP deficiency. J Clin Immunol. (2021) 41:1975–8. doi: 10.1007/s10875-021-01139-8, PMID: 34580798

[39] Iacobaeus E Burkill S Bahmanyar S Hakim R Bystrom C Fored M. The national incidence of PML in Sweden, 1988-2013. Neurology. (2018) 90:e498–506. doi: 10.1212/WNL.0000000000004926, PMID: 29321229 PMC5818018

[40] Joly M Conte C Cazanave C Le Moing V Tattevin P Delobel P. Progressive multifocal leukoencephalopathy: epidemiology and spectrum of predisposing conditions. Brain. (2023) 146:349–58. doi: 10.1093/brain/awac237, PMID: 35779271

[41] Engel P Eck MJ Terhorst C. The SAP and SLAM families in immune responses and X-linked lymphoproliferative disease. Nat Rev Immunol. (2003) 3:813–21. doi: 10.1038/nri1202, PMID: 14523387

[42] Ma CS Nichols KE Tangye SG. Regulation of cellular and humoral immune responses by the SLAM and SAP families of molecules. Annu Rev Immunol. (2007) 25:337–79. doi: 10.1146/annurev.immunol.25.022106.141651, PMID: 17201683

[43] Angulo A Cuenca M Martinez-Vicente P Engel P. Viral CD229 (Ly9) homologs as new manipulators of host immunity. J Leukoc Biol. (2019) 105:947–54. doi: 10.1002/JLB.2MR1018-413R, PMID: 30791129

[44] van Driel BJ Liao G Engel P Terhorst C. Responses to microbial challenges by SLAMF receptors. Front Immunol. (2016) 7:4. doi: 10.3389/fimmu.2016.00004, PMID: 26834746 PMC4718992

[45] Volkmer B Planas R Gossweiler E Lunemann A Opitz L Mauracher A. Recurrent inflammatory disease caused by a heterozygous mutation in CD48. J Allergy Clin Immunol. (2019) 144:1441–1445.e17. doi: 10.1016/j.jaci.2019.07.038, PMID: 31419545

[46] Fouquet G Marcq I Debuysscher V Bayry J Rabbind Singh A Bengrine A. Signaling lymphocytic activation molecules Slam and cancers: friends or foes? Oncotarget. (2018) 9:16248–62. doi: 10.18632/oncotarget.24575, PMID: 29662641 PMC5882332

[47] Ishibashi M Morita R Tamura H. Immune functions of signaling lymphocytic activation molecule family molecules in multiple myeloma. Cancers (Basel). (2021) 13:279. doi: 10.3390/cancers13020279, PMID: 33451089 PMC7828503

[48] Gartshteyn Y Askanase AD Mor A. SLAM associated protein signaling in T cells: tilting the balance toward autoimmunity. Front Immunol. (2021) 12:654839. doi: 10.3389/fimmu.2021.654839, PMID: 33936082 PMC8086963

[49] Moser KL Neas BR Salmon JE Yu H Gray-McGuire C Asundi N. Genome scan of human systemic lupus erythematosus: evidence for linkage on chromosome 1q in African-American pedigrees. Proc Natl Acad Sci USA. (1998) 95:14869–74. doi: 10.1073/pnas.95.25.14869, PMID: 9843982 PMC24542

[50] Wandstrat AE Nguyen C Limaye N Chan AY Subramanian S Tian XH. Association of extensive polymorphisms in the SLAM/CD2 gene cluster with murine lupus. Immunity. (2004) 21:769–80. doi: 10.1016/j.immuni.2004.10.009, PMID: 15589166

[51] Cunninghame Graham DS Vyse TJ Fortin PR Montpetit A Cai YC Lim S. Association of LY9 in UK and Canadian SLE families. Genes Immun. (2008) 9:93–102. doi: 10.1038/sj.gene.6364453, PMID: 18216865

[52] Humbel M Bellanger F Horisberger A Suffiotti M Fluder N Makhmutova M. SLAMF receptor expression identifies an immune signature that characterizes systemic lupus erythematosus. Front Immunol. (2022) 13:843059. doi: 10.3389/fimmu.2022.843059, PMID: 35603218 PMC9120573

[53] Karampetsou MP Comte D Kis-Toth K Kyttaris VC Tsokos GC. Expression patterns of signaling lymphocytic activation molecule family members in peripheral blood mononuclear cell subsets in patients with systemic lupus erythematosus. PLoS One. (2017) 12:e0186073. doi: 10.1371/journal.pone.0186073, PMID: 29020082 PMC5636110

[54] Liu Q Deng Y Liu X Zheng Y Li Q Cai G. Transcriptomic analysis of B cells suggests that CD70 and LY9 may be novel features in patients with systemic lupus erythematosus. Heliyon. (2023) 9:e15684. doi: 10.1016/j.heliyon.2023.e15684, PMID: 37144201 PMC10151360

[55] Szklarczyk D Nastou K Koutrouli M Kirsch R Mehryary F Hachilif R. The STRING database in 2025: protein networks with directionality of regulation. Nucleic Acids Res. (2025) 53:D730–7. doi: 10.1093/nar/gkae1113, PMID: 39558183 PMC11701646

[56] Abdalgani M Hernandez ER Pedroza LA Chinn IK Forbes Satter LR Rider NL. Clinical, immunologic, and genetic characteristics of 148 patients with natural killer cell deficiency. J Allergy Clin Immunol. (2025) 155:1623–34. doi: 10.1016/j.jaci.2025.01.030, PMID: 39914554 PMC12058391

[57] Mace EM Orange JS. Emerging insights into human health and NK cell biology from the study of NK cell deficiencies. Immunol Rev. (2019) 287:202–25. doi: 10.1111/imr.12725, PMID: 30565241 PMC6310041

[58] Mace EM. Human natural killer cells: form, function, and development. J Allergy Clin Immunol. (2023) 151:371–85. doi: 10.1016/j.jaci.2022.09.022, PMID: 36195172 PMC9905317

[59] Vivier E Raulet DH Moretta A Caligiuri MA Zitvogel L Lanier LL. Innate or adaptive immunity? The example of natural killer cells. Science. (2011) 331:44–9. doi: 10.1126/science.1198687, PMID: 21212348 PMC3089969

[60] Shu X Gao X Dai Y Wang Y Liu Y Wang D. C3 as a predictive and prognostic biomarker in adult hemophagocytic lymphohistiocytosis: a large cohort study in China. Blood Adv. (2025) 9:1836–46. doi: 10.1182/bloodadvances.2024014715, PMID: 39913689 PMC12008621

[61] Gloude NJ Dandoy CE Davies SM Myers KC Jordan MB Marsh RA. Thinking beyond HLH: clinical features of patients with concurrent presentation of Hemophagocytic Lymphohistiocytosis and thrombotic Microangiopathy. J Clin Immunol. (2020) 40:699–707. doi: 10.1007/s10875-020-00789-4, PMID: 32447592 PMC7245179

[62] Minoia F Tibaldi J Muratore V Gallizzi R Bracaglia C Arduini A. Thrombotic Microangiopathy associated with macrophage activation syndrome: a multinational study of 23 patients. J Pediatr. (2021) 235:196–202. doi: 10.1016/j.jpeds.2021.04.004, PMID: 33836183

[63] Atkinson AL Atwood WJ. Fifty years of JC polyomavirus: a brief overview and remaining questions. Viruses. (2020) 12:969. doi: 10.3390/v12090969, PMID: 32882975 PMC7552028

[64] Butic AB Spencer SA Shaheen SK Lukacher AE. Polyomavirus wakes up and chooses neurovirulence. Viruses. (2023) 15:2112. doi: 10.3390/v15102112, PMID: 37896889 PMC10612099

[65] Darbinyan A Siddiqui KM Slonina D Darbinian N Amini S White MK. Role of JC virus agnoprotein in DNA repair. J Virol. (2004) 78:8593–600. doi: 10.1128/JVI.78.16.8593-8600.2004, PMID: 15280468 PMC479055

[66] Yue X Bai C Xie D Ma T Zhou PK. DNA-PKcs: a multi-faceted player in DNA damage response. Front Genet. (2020) 11:607428. doi: 10.3389/fgene.2020.607428, PMID: 33424929 PMC7786053

[67] Darbinyan A White MK Akan S Radhakrishnan S Del Valle L Amini S. Alterations of DNA damage repair pathways resulting from JCV infection. Virology. (2007) 364:73–86. doi: 10.1016/j.virol.2007.02.015, PMID: 17368705 PMC2570112

[68] Trojanek J Croul S Ho T Wang JY Darbinyan A Nowicki M. T-antigen of the human polyomavirus JC attenuates faithful DNA repair by forcing nuclear interaction between IRS-1 and rad 51. J Cell Physiol. (2006) 206:35–46. doi: 10.1002/jcp.20425, PMID: 15965906

[69] Suzuki T Orba Y Makino Y Okada Y Sunden Y Hasegawa H. Viroporin activity of the JC polyomavirus is regulated by interactions with the adaptor protein complex 3. Proc Natl Acad Sci USA. (2013) 110:18668–73. doi: 10.1073/pnas.1311457110, PMID: 24167297 PMC3832026

[70] Saribas AS Datta PK Safak M. A comprehensive proteomics analysis of JC virus Agnoprotein-interacting proteins: Agnoprotein primarily targets the host proteins with coiled-coil motifs. Virology. (2020) 540:104–18. doi: 10.1016/j.virol.2019.10.005, PMID: 31765920 PMC6957716

[71] Strazic Geljic I Kucan Brlic P Musak L Karner D Ambriovic-Ristov A Jonjic S. Viral interactions with adaptor-protein complexes: a ubiquitous trait among viral species. Int J Mol Sci. (2021) 22:5274. doi: 10.3390/ijms22105274, PMID: 34067854 PMC8156722

[72] Deffner M Schneider-Hohendorf T Schulte-Mecklenbeck A Falk S Lu IN Ostkamp P. Chemokine-mediated cell migration into the central nervous system in progressive multifocal leukoencephalopathy. Cell Rep Med. (2024) 5:101622. doi: 10.1016/j.xcrm.2024.101622, PMID: 38917802 PMC11293326

[73] Schweitzer F Laurent S Cortese I Fink GR Silling S Skripuletz T. Progressive multifocal leukoencephalopathy: pathogenesis, diagnostic tools, and potential biomarkers of response to therapy. Neurology. (2023) 101:700–13. doi: 10.1212/WNL.0000000000207622, PMID: 37487750 PMC10585672

[74] Wortman MJ Lundberg PS Dagdanova AV Venkataraman P Daniel DC Johnson EM. Opportunistic DNA recombination with Epstein-Barr virus at sites of control region rearrangements mediating JC virus Neurovirulence. J Infect Dis. (2016) 213:1436–43. doi: 10.1093/infdis/jiv755, PMID: 26690342 PMC4813741

[75] Barbier MT Del Valle L. Co-detection of EBV and human polyomavirus JCPyV in a case of AIDS-related multifocal primary central nervous system diffuse large B-cell lymphoma. Viruses. (2023) 15:755. doi: 10.3390/v15030755, PMID: 36992464 PMC10059075

[76] Bennett CL Focosi D Socal MP Bian JC Nabhan C Hrushesky WJ. Progressive multifocal leukoencephalopathy in patients treated with rituximab: a 20-year review from the southern network on adverse reactions. Lancet Haematol. (2021) 8:e593–604. doi: 10.1016/S2352-3026(21)00167-8, PMID: 34329579

[77] Brancati S Gozzo L Longo L Vitale DC Drago F. Rituximab in multiple sclerosis: are we ready for regulatory approval? Front Immunol. (2021) 12:661882. doi: 10.3389/fimmu.2021.661882, PMID: 34295328 PMC8290177

[78] Sarsour K Beckley-Kartey S Melega S Odueyungbo A Kirchner P Khalife N. Rituximab utilization for approved and off-label nononcology indications and patients' experiences with the patient alert card. Pharmacol Res Perspect. (2020) 8:e00555. doi: 10.1002/prp2.555, PMID: 31911839 PMC6941895

[79] Alatawi YM Hansen RA. Empirical estimation of under-reporting in the U.S. Food and Drug Administration adverse event reporting system (FAERS). Expert Opin Drug Saf. (2017) 16:761–7. doi: 10.1080/14740338.2017.1323867, PMID: 28447485

[80] Hazell L Shakir SA. Under-reporting of adverse drug reactions: a systematic review. Drug Saf. (2006) 29:385–96. doi: 10.2165/00002018-200629050-00003, PMID: 16689555

[81] Sakaeda T Tamon A Kadoyama K Okuno Y. Data mining of the public version of the FDA adverse event reporting system. Int J Med Sci. (2013) 10:796–803. doi: 10.7150/ijms.6048, PMID: 23794943 PMC3689877

[82] Foley JF Defer G Ryerson LZ Cohen JA Arnold DL Butzkueven H. Pharmacokinetics and pharmacodynamics of Natalizumab 6-week dosing vs continued 4-week dosing for relapsing-remitting multiple sclerosis. Neurol Neuroimmunol Neuroinflamm. (2024) 11:e200321. doi: 10.1212/NXI.0000000000200321, PMID: 39393045 PMC11488827

[83] Ryerson LZ Foley J Chang I Kister I Cutter G Metzger RR. Risk of natalizumab-associated PML in patients with MS is reduced with extended interval dosing. Neurology. (2019) 93:e1452–62. doi: 10.1212/WNL.0000000000008243, PMID: 31515290 PMC7010325

[84] Ikumi K Ando T Katano H Katsuno M Sakai Y Yoshida M. HSV-2-related hemophagocytic lymphohistiocytosis in a fingolimod-treated patient with MS. Neurol Neuroimmunol Neuroinflamm. (2016) 3:e247. doi: 10.1212/NXI.0000000000000247, PMID: 27308307 PMC4897984

[85] Machlańska A Helbig G Chromik K Zapała M Zwiernik B Selmaj K. Hemophagocytic lymphohistiocytosis associated with ocrelizumab treatment in a patient with multiple sclerosis. Mult Scler. (2021) 27:1803–5. doi: 10.1177/1352458521993070, PMID: 33666121

[86] Ošep AB Brecl E Skerget M Savsek L. An unforeseen reality: Hemophagocytic lymphohistiocytosis following alemtuzumab treatment for a multiple sclerosis. Clin Neurol Neurosurg. (2023) 228:107675. doi: 10.1016/j.clineuro.2023.107675, PMID: 36965418

[87] Romero A Midaglia L Salcedo MT Viladomiu L Guillen E Bajana I. Hemophagocytic syndrome following alemtuzumab treatment for multiple sclerosis: a case report. Mult Scler Relat Disord. (2020) 40:101973. doi: 10.1016/j.msard.2020.101973, PMID: 32028116

[88] Saarela M Senthil K Jones J Tienari PJ Soilu-Hanninen M Airas L. Hemophagocytic lymphohistiocytosis in 2 patients with multiple sclerosis treated with alemtuzumab. Neurology. (2018) 90:849–51. doi: 10.1212/WNL.0000000000005420, PMID: 29602914

[89] Smulski CR Eibel H. BAFF and BAFF-receptor in B cell selection and survival. Front Immunol. (2018) 9:2285. doi: 10.3389/fimmu.2018.02285, PMID: 30349534 PMC6186824

[90] Hoeynck BW Cohen AD Stadtmauer EA Susanibar-Adaniya SP Vogl DT Waxman AJ. Progressive multifocal leukoencephalopathy in multiple myeloma. Eur J Haematol. (2023) 110:322–9. doi: 10.1111/ejh.13909, PMID: 36465014

[91] Koutsavlis I. Progressive multifocal leukoencephalopathy in multiple myeloma. A literature review and lessons to learn. Ann Hematol. (2021) 100:1–10. doi: 10.1007/s00277-020-04294-x, PMID: 33009935

[92] Usui K Kitazaki Y Enomoto S Morita M Nakamichi K Hamano T. A case of progressive multifocal leukoencephalopathy associated with daratumumab, bortezomib, and dexamethasone for multiple myeloma. Rinsho Shinkeigaku. (2023) 63:513–7. doi: 10.5692/clinicalneurol.cn-001847, PMID: 37518017

[93] Usui Y Nakano H Komatsu J Nakamichi K Saijo M Takano S. Progressive multifocal leukoencephalopathy during treatment with lenalidomide and elotuzumab for multiple myeloma. Leuk Lymphoma. (2020) 61:2234–7. doi: 10.1080/10428194.2020.1765237, PMID: 32420767

[94] Berger JR Hartung HP. Commentary: progressive multifocal leukoencephalopathy genetic risk variants for pharmacovigilance of immunosuppressant therapies. Front Neurol. (2023) 14:1146027. doi: 10.3389/fneur.2023.1146027, PMID: 37006492 PMC10062523

[95] Pacanowski M Schuck RN. Evidence, in context: a regulatory perspective on Pharmacogenetics. Clin Pharmacol Ther. (2022) 111:1202–4. doi: 10.1002/cpt.2347, PMID: 34278575

[96] Adelon J Abolhassani H Esenboga S Fouyssac F Cagdas D Tezcan I. Human DNA-dependent protein kinase catalytic subunit deficiency: a comprehensive review and update. J Allergy Clin Immunol. (2024) 154:1300–12. doi: 10.1016/j.jaci.2024.06.018, PMID: 38977084

[97] Al Shekaili L Sheikh F Al Gazlan S Al Dhekri H Al Mousa H Al Ghonaium A. Novel mutation in DOCK8-HIES with severe phenotype and successful transplantation. Clin Immunol. (2017) 178:39–44. doi: 10.1016/j.clim.2016.08.002, PMID: 27890707

[98] Aschermann Z Gomori E Kovacs GG Pal E Simon G Komoly S. X-linked hyper-IgM syndrome associated with a rapid course of multifocal leukoencephalopathy. Arch Neurol. (2007) 64:273–6. doi: 10.1001/archneur.64.2.273, PMID: 17296845

[99] Bahrami S Arshi S Nabavi M Bemanian MH Fallahpour M Rezaeifar A. Progressive multifocal leukoencephalopathy in a patient with novel mutation in the RAC2 gene: a case report. J Med Case Rep. (2022) 16:235. doi: 10.1186/s13256-022-03333-7, PMID: 35689244 PMC9188039

[100] Day-Williams AG Sun C Jelcic I McLaughlin H Harris T Martin R. Whole genome sequencing reveals a chromosome 9p deletion causing DOCK8 deficiency in an adult diagnosed with hyper IgE syndrome who developed progressive multifocal leukoencephalopathy. J Clin Immunol. (2015) 35:92–6. doi: 10.1007/s10875-014-0114-4, PMID: 25388448 PMC4306731

[101] Delmonte OM Bergerson JRE Kawai T Kuehn HS McDermott DH Cortese I. SASH3 variants cause a novel form of X-linked combined immunodeficiency with immune dysregulation. Blood. (2021) 138:1019–33. doi: 10.1182/blood.2020008629, PMID: 33876203 PMC8462359

[102] Dhalla F Murray S Sadler R Chaigne-Delalande B Sadaoka T Soilleux E. Identification of a novel mutation in MAGT1 and progressive multifocal leucoencephalopathy in a 58-year-old man with XMEN disease. J Clin Immunol. (2015) 35:112–8. doi: 10.1007/s10875-014-0116-2, PMID: 25504528 PMC6328310

[103] Dobbs K Tabellini G Calzoni E Patrizi O Martinez P Giliani SC. Natural killer cells from patients with recombinase-activating gene and non-homologous end joining gene defects comprise a higher frequency of CD56^bright^ NKG2A^+++^ cells, and yet display increased degranulation and higher Perforin content. Front Immunol. (2017) 8:798. doi: 10.3389/fimmu.2017.00798, PMID: 28769923 PMC5511964

[104] Donadieu J Lamant M Fieschi C de Fontbrune FS Caye A Ouachee M. Natural history of GATA2 deficiency in a survey of 79 French and Belgian patients. Haematologica. (2018) 103:1278–87. doi: 10.3324/haematol.2017.181909, PMID: 29724903 PMC6068047

[105] Downes SM Black GC Hyman N Simmonds M Morris J Barton C. Visual loss due to progressive multifocal leukoencephalopathy in a congenital immunodeficiency disorder. Arch Ophthalmol. (2001) 119:1376–8. doi: 10.1001/archopht.119.9.1376, PMID: 11545648

[106] Durkee-ShockJZhangALiangHWrightHMagnussonJGarabedianE. Morbidity, mortality, and therapeutics in combined immunodeficiency: data from the USIDNET registry. J Allergy Clin Immunol Pract. (2022) 10:1334–1341.e6. doi: 10.1016/j.jaip.2022.01.042, PMID: 35172220

[107] Emmanouilidou E Kosmara D Papadaki E Mastorodemos V Constantoulakis P Repa A. Progressive multifocal leukoencephalopathy in systemic lupus erythematosus: a consequence of patient-intrinsic or -extrinsic factors? J Clin Med. (2023) 12:6945. doi: 10.3390/jcm12216945, PMID: 37959410 PMC10647998

[108] Engelhardt KR Gertz ME Keles S Schaffer AA Sigmund EC Glocker C. The extended clinical phenotype of 64 patients with dedicator of cytokinesis 8 deficiency. J Allergy Clin Immunol. (2015) 136:402–12. doi: 10.1016/j.jaci.2014.12.1945, PMID: 25724123 PMC4530066

[109] Engelhardt KR McGhee S Winkler S Sassi A Woellner C Lopez-Herrera G. Large deletions and point mutations involving the dedicator of cytokinesis 8 (DOCK8) in the autosomal-recessive form of hyper-IgE syndrome. J Allergy Clin Immunol. (2009) 124:1289–1302.e4. doi: 10.1016/j.jaci.2009.10.038, PMID: 20004785 PMC2818862

[110] Hadjadj J Guffroy A Delavaud C Taieb G Meyts I Fresard A. Progressive multifocal leukoencephalopathy in primary Immunodeficiencies. J Clin Immunol. (2019) 39:55–64. doi: 10.1007/s10875-018-0578-8, PMID: 30552536

[111] Hernandez-Trujillo V Zhou C Scalchunes C Ochs HD Sullivan KE Cunningham-Rundles C. A registry study of 240 patients with X-linked Agammaglobulinemia living in the USA. J Clin Immunol. (2023) 43:1468–77. doi: 10.1007/s10875-023-01502-x, PMID: 37219739 PMC10354121

[112] Le Voyer T Maglorius Renkilaraj MRL Moriya K Perez Lorenzo M Nguyen T Gao L. Inherited human Rel B deficiency impairs innate and adaptive immunity to infection. Proc Natl Acad Sci USA. (2024) 121:e2321794121. doi: 10.1073/pnas.2321794121, PMID: 39231201 PMC11406260

[113] Le Voyer T Parent AV Liu X Cederholm A Gervais A Rosain J. Autoantibodies against type I IFNs in humans with alternative NF-kappa B pathway deficiency. Nature. (2023) 623:803–13. doi: 10.1038/s41586-023-06717-x, PMID: 37938781 PMC10665196

[114] Li Q Tang C Zhu J Zhang L. A case of progressive multifocal leukoencephalopathy with hypogammaglobulinemia and a TCF3 mutation. J Neurovirol. (2022) 28:616–8. doi: 10.1007/s13365-022-01092-1, PMID: 35976539

[115] Lorenzini T Fliegauf M Klammer N Frede N Proietti M Bulashevska A. Characterization of the clinical and immunologic phenotype and management of 157 individuals with 56 distinct heterozygous NFKB1 mutations. J Allergy Clin Immunol. (2020) 146:901–11. doi: 10.1016/j.jaci.2019.11.051, PMID: 32278790 PMC8246418

[116] MacDougall M Afzal S Jiang S McGhee S Lewis D. A case of inducible T-cell co-stimulator ligand deficiency with severe viral infection and autoimmune hepatitis. Ann Allergy Asthma Immunol. (2024) 133:S179–80. doi: 10.1016/j.anai.2024.08.698

[117] Maffucci P Filion CA Boisson B Itan Y Shang L Casanova JL. Genetic diagnosis using whole exome sequencing in common variable immunodeficiency. Front Immunol. (2016) 7:220. doi: 10.3389/fimmu.2016.00220, PMID: 27379089 PMC4903998

[118] Mørup SB Nazaryan-Petersen L Gabrielaite M Reekie J Marquart HV Hartling HJ. Added value of reanalysis of whole exome- and whole genome sequencing data from patients suspected of primary immune deficiency using an extended gene panel and structural variation calling. Front Immunol. (2022) 13:906328. doi: 10.3389/fimmu.2022.906328, PMID: 35874679 PMC9302041

[119] Nademi Z Wynn RF Slatter M Hughes SM Bonney D Qasim W. Hematopoietic stem cell transplantation for cytidine triphosphate synthase 1 (CTPS1) deficiency. Bone Marrow Transplant. (2019) 54:130–3. doi: 10.1038/s41409-018-0246-x, PMID: 29884857

[120] Nitta H Unoki M Ichiyanagi K Kosho T Shigemura T Takahashi H. Three novel ZBTB24 mutations identified in Japanese and cape Verdean type 2 ICF syndrome patients. J Hum Genet. (2013) 58:455–60. doi: 10.1038/jhg.2013.56, PMID: 23739126

[121] Sampaio EP Hsu AP Pechacek J Bax HI Dias DL Paulson ML. Signal transducer and activator of transcription 1 (STAT1) gain-of-function mutations and disseminated coccidioidomycosis and histoplasmosis. J Allergy Clin Immunol. (2013) 131:1624–1634.e17. doi: 10.1016/j.jaci.2013.01.052, PMID: 23541320 PMC3746066

[122] Schröder C Baerlecken NT Pannicke U Dörk T Witte T Jacobs R. Evaluation of RAG1 mutations in an adult with combined immunodeficiency and progressive multifocal leukoencephalopathy. Clin Immunol. (2017) 179:1–7. doi: 10.1016/j.clim.2016.12.013, PMID: 28216420

[123] Schwartzmann Y Vaknin-Dembinsky A Gomori JM Elinav H Berkun Y Levin N. Tofacitinib-induced progressive multifocal leukoencephalopathy-immune reconstitution inflammatory syndrome. Neurol Sci. (2023) 44:3737–9. doi: 10.1007/s10072-023-06897-4, PMID: 37306796

[124] Suzuki H Takahashi Y Miyajima H. Progressive multifocal leukoencephalopathy complicating X-linked hyper-IgM syndrome in an adult. Intern Med. (2006) 45:1187–8. doi: 10.2169/internalmedicine.45.6023, PMID: 17106168

[125] Teramoto T Kaneko H Funato M Sawa H Nagashima K Hirose Y. Progressive multifocal leukoencephalopathy in a patient with X-linked agammaglobulinemia. Scand J Infect Dis. (2003) 35:909–10. doi: 10.1080/00365540310016673, PMID: 14723382

[126] Tuijnenburg P Lango Allen H Burns SO Greene D Jansen MH Staples E. Loss-of-function nuclear factor kappa B subunit 1 (NFKB1) variants are the most common monogenic cause of common variable immunodeficiency in Europeans. J Allergy Clin Immunol. (2018) 142:1285–96. doi: 10.1016/j.jaci.2018.01.039 29477724 PMC6148345

[127] Volk T Warnatz K Marks R Urbach H Schluh G Strohmeier V. Pembrolizumab for treatment of progressive multifocal leukoencephalopathy in primary immunodeficiency and/or hematologic malignancy: a case series of five patients. J Neurol. (2022) 269:973–81. doi: 10.1007/s00415-021-10682-8, PMID: 34196768 PMC8782776

[128] Almeida DPM Reis B Faccion RS Cunha D Agonigi B Machado L. Novel non-coding variant in DCLRE1C segregates with distinct severe combined immunodeficiency phenotypes and Hodgkin lymphoma in consanguineous siblings. Hematol Transfus Cell Ther. (2024) 46:S208–9. doi: 10.1016/j.htct.2024.09.349

[129] Agrawal P Sharma S Pal P Ojha H Mullick J Sahu A. The imitation game: a viral strategy to subvert the complement system. FEBS Lett. (2020) 594:2518–42. doi: 10.1002/1873-3468.13856, PMID: 32506518

[130] Kolev M Le Friec G Kemper C. Complement--tapping into new sites and effector systems. Nat Rev Immunol. (2014) 14:811–20. doi: 10.1038/nri3761, PMID: 25394942

[131] Mayilyan KR. Complement genetics, deficiencies, and disease associations. Protein Cell. (2012) 3:487–96. doi: 10.1007/s13238-012-2924-6, PMID: 22773339 PMC4875391

[132] Murugaiah V Varghese PM Beirag N De Cordova S Sim RB Kishore U. Complement proteins as soluble pattern recognition receptors for pathogenic viruses. Viruses. (2021) 13:824. doi: 10.3390/v13050824, PMID: 34063241 PMC8147407

[133] Mastellos DC Hajishengallis G Lambris JD. A guide to complement biology, pathology and therapeutic opportunity. Nat Rev Immunol. (2024) 24:118–41. doi: 10.1038/s41577-023-00926-1, PMID: 37670180

[134] Planas R Felber M Vavassori S Pachlopnik Schmid J. The hyperinflammatory spectrum: from defects in cytotoxicity to cytokine control. Front Immunol. (2023) 14:1163316. doi: 10.3389/fimmu.2023.1163316, PMID: 37187762 PMC10175623

[135] Mastio J Saeed MB Wurzer H Krecke M Westerberg LS Thomas C. Higher incidence of B cell malignancies in primary Immunodeficiencies: a combination of intrinsic genomic instability and exocytosis defects at the immunological synapse. Front Immunol. (2020) 11:581119. doi: 10.3389/fimmu.2020.581119, PMID: 33240268 PMC7680899

[136] Riaz IB Faridi W Patnaik MM Abraham RS. A systematic review on predisposition to lymphoid (B and T cell) Neoplasias in patients with primary Immunodeficiencies and immune Dysregulatory disorders (inborn errors of immunity). Front Immunol. (2019) 10:777. doi: 10.3389/fimmu.2019.00777, PMID: 31057537 PMC6477084

