# Supplementary material for: Genetics of progressive multifocal leukoencephalopathy: update on case reports with an inborn error of immunity and risk variants found in drug-linked cases
Source: Front Neurol. 2025 Jul 15;16:1629581. doi: 10.3389/fneur.2025.1629581 (PMC12320240; doi:10.3389/fneur.2025.1629581)
Supplement: Supplementary file 1 [file Data_Sheet_1.pdf]

SUPPLEMENTARY TABLE 1 | Genetic information for previously reported progressive multifocal leukoencephalopathy (PML) case reports diagnosed with an inborn error of immunity (IEI) or cases carrying a PML risk variant.[a]

PML case ID	Country of origin	Sex	IEI table #[b]	IEI disease (based on impacted gene)	MIM gene	MIM phenotype	IEI onset age (yr)	PML onset age (yr)	Death age (yr)/ outcome	Gene symbol (HGNC)	Gene symbol aliase (IUIS)	RefSeq transcript variant	Disease model	Inheritance	Variant (hg38)	Variant, HGVSc	Variant, HGVSp or consequence	gnomAD 4.1.0 AF (Total)[c]	Other immune conditions	IS drugs used	PML case reference
**Patients diagnosed with an IEI and PML**																					
1A	Japan	M	3.1	BTK deficiency, X-linked agammaglobulinemia (XLA)	300300	300755	6	37	38	*BTK*		NM_000061.3	XL	hemi	X-101358670-T-C	c.921A>G	p.Arg307Ser	0	n/a	n/a	Teramoto et al. 2003 (125)
1B	USA	n/a	3.1				n/a	n/a	n/a					n/a	n/a	n/a	n/a	n/a	n/a	n/a	Hernandez-Trujillo et al. 2023 (111)
2A	Japan	M	1.3	CD40 ligand (CD154) deficiency	300386	308230	n/a	37	n/a	*CD40LG*	*CD154, CD40L*	NM_000074.3	XL	hemi	X-136659303-T-C	c.674T>C	p.Leu225Ser	0	n/a	n/a	Suzuki et al. 2006 (124)
2B	Hungary	M	1.3				3	19	19					hemi	X-136650325-C-A	c.216C>A	p.Cys72Ter	0	neutropenia	n/a	Aschermann et al. 2007 (98)
2C	n/a	M	1.3				11	35	35					hemi	n/a	n/a	n/a	n/a	arthritis, neutropenia	n/a	Hadjadj et al. 2018 (110)
2D	n/a	M	1.3				n/a	21	n/a					hemi	n/a	n/a	n/a	n/a	n/a	n/a	Volk et al. 2022 (127)
2E	USA	n/a	1.3				11	n/a	17					hemi	n/a	n/a	n/a	n/a	see case report	n/a	Durkee-Shock et al. 2022 (106)
3A	USA	n/a	4.3	CTLA4 haploinsufficiency (ALPS-V)	123890	616100	36	n/a	40	*CTLA4*		NM_005214.5	AD	n/a	n/a	n/a	n/a	n/a	see case report	prednisone, sirolimus	Durkee-Shock et al. 2022 (106)
4A	UK	M	4.7	CTPS1 deficiency	123860	615897	1	13	14	*CTPS1*		NM_001905.4	AR	hom	1-41010160-G-C	c.1692-1G>C	LOF (splice acceptor)	5.64E-04	see case report	alemtuzumab, fludarabine	Nademi et al. 2018 (119)
5A	n/a	n/a	1.2	DCLRE1C (Artemis) deficiency	605988	602450	12	n/a	n/a	*DCLRE1C*	*ARTEMIS*	NM_001033855.3	AR	hom	n/a	n/a	p.Ser147fs*6[d]	n/a	vasculitis	n/a	Dobbs et al. 2017 (103)
6A	Finland	M	2.9	DIAPH1 deficiency	602121	616632	13	29	30	*DIAPH1*		NM_005219.5	AR	hom	5-141582311-C-T	c.684+1G>A	LOF (splice donor)	3.29E-05	lymphopenia, skin ulcers	n/a	Kaustio et al. 2021 (20)
7A	Italy	M	1.3	DOCK8 deficiency	611432	243700	8	8	8	*DOCK8*		NM_203447.4	AR	comp het	n/a	EX19_45del;<br>EX?(early exon)_48del	LOF<br>LOF	n/a<br>n/a	eczema, food allergies	n/a	Engelhardt et al. 2009 & 2015 (108, 109)
7B	Turkey	M	1.3				6	6	6					hom	n/a	EX?(3)_25del	LOF	n/a	eczema, food allergies	n/a	Engelhardt et al. 2009 & 2015 (108, 109)
7C	n/a	M	1.3				6	30	n/a					hom	9-20211-596751_del	whole gene*	LOF	n/a	eczema	corticosteroids	Day-Williams et al. 2014 (100)
7D	Iran	M	1.3				8	8	8					hom	n/a	EX11_14del	LOF	n/a	eczema, food allergy	n/a	Engelhardt et al. 2015 (108)
7E	Saudia Arabia	M	1.3				5	16	16					hom	9-428369-C-T;<br>9-429859-G-A	c.4346C>T;<br>c.4626+5G>A	p.Ser1449Leu;<br>n/a	1.02E-04;<br>0	eczema and see case report	n/a	Al Shekaili et al. 2016 (97)
7F	n/a	F	1.3				5	15	16					comp het	n/a	2 large het del	LOF	n/a	vulvar cancer	n/a	Hadjadj et al. 2018 (110)
7G	n/a	M	1.3				n/a	45	45					comp het	n/a	n/a	n/a	n/a	lymphopenia, skin tumors	n/a	Volk et al. 2022 (127)
8A	Belgium/France	n/a	5.4	GATA2 deficiency	137295	614172	43	43	43	*GATA2*		NM_032638.5	AD	het	3-128481857-CAG-G	c.1103_1104del	p.Pro368Argfs15	0	n/a	n/a	Donadieu et al. 2018 (104)
8B[e]	n/a	F	5.4				n/a	33	34					het	3-128481830-T-C	c.1132A>G	p.Lys378Glu	0	SLE, hypothyroidism	azathioprine, belimumab, cyclosporine,<br>methotrexate, prednisolone	Emmanouilidou et al. 2023 (107)
9A	USA	M	1.3	ICOSL deficiency	605717	620825	n/a	46	alive	*ICOSLG*	*ICOSL*	NM_015259.6	AR	hom	21-44238447-C-A	c.55+1G>T	LOF (splice donor)	0	see case report	sirolimus	MacDougall et al. 2024 (116)
10A	USA	n/a	2.7 or<br>7.3	EDA-ID due to NEMO/IKBKG deficiency<br>NEMO exon 5 deletion	300248	300291, 300636<br>301081	< 1	n/a	32	*IKBKG*	*NEMO*	n/a	XL	hemi	n/a	n/a	n/a	n/a	encephalopathy	n/a	Durkee-Shock et al. 2022 (106)
11A	UK	M	4.7	X-linked magnesium EBV neoplasia (XMEN)	300715	300853	36	58	58	*MAGT1*		NM_001367916.1	XL	hemi	X-77856789-G-A	c.616C>T	p.Arg206Ter	0	see case report	CHOP, rituximab	Dhalla et al. 2014 (102)
12A	USA	F	3.2	NFKB1 deficiency	164011	616576	18	48	48	*NFKB1*		NM_003998.4	AD	het	4-102584711-T-A	c.957T>A	p.Tyr319Ter	0	see case report	n/a	Maffucci et al. 2016 (117)
12B	Belgium	M	3.2				5	20	alive					het	4-102582880-C-T	c.850C>T	p.Arg284Ter	0	n/a	n/a	Tuijnenburg et al. 2018 (126)
12C	Germany	M	3.2				10	n/a	alive					het	4-102576937-C-T	c.469C>T	p.Arg157Ter	0	see case report	corticosteroids	Lorenzini et al. 2020 (115)
13A	France	F	3.2	NFKB2 deficiency	164012	615577	9	n/a	died (age n/a)	*NFKB2*		NM_001322934.2	AD	het	10-102402138-C-T	c.2557C>T	p.Arg853Ter	0	see case report	n/a	Le Voyer et al. 2023 (113)
14A	Turkey	F	1.2	DNA PKcs deficiency	600899	615966	< 1	22	23	*PRKDC*	*DNA-PKcs*	NM_006904.7	AR	hom	[8-47858853-TCTC-T;<br>8-47820870-A-C]	c.[6338_6340del;<br>9185T>G]	p.[Gly2113del;<br>Leu3062Arg]	3.16E-03;<br>0	arthritis, DLBCL, and<br>see case report	methotrexate, rituximab (R-CHOP)	Hadjadj et al. 2018, Adelon et al. 2024 (96, 110)
15A	Iran	M	3.2	RAC2 deficiency	602049	618987	2	34	34	*RAC2*		NM_002872.5	AR	hom	22-37232859-C-T	c.167G>A	p.Trp56Ter	0	see case report	n/a	Bahrami et al. 2022 (99)
16A	Germany	M	1.2	RAG deficiency	179615	601457	35	37	37	*RAG1*		NM_000448.3	AR	comp het	[11-36574427-C-G;<br>11-36574734-CC-C];<br>11-36574724-C-T	c.[1123C>G;<br>1430delC];<br>c.1420C>T	p.[His375Asp;<br>Phe478SerfsTer14];<br>p.Arg474Cys	5.58E-06;<br>0;<br>4.03E-05	see case report	n/a	Schröder et al. 2017 (122)
16B	Denmark	F	1.2				< 10	n/a	died (age n/a)				AD	het	11-36573782-C-T	c.478C>T	p.Arg160Trp	9.29E-06	see case report	n/a	Mørup et al. 2022 (118)
17A	Canada	M	1.3	RELB deficiency	604758	617585	< 1	n/a	34	*RELB*		NM_006509.4	AR	comp het	19-45012205-G-A;<br>19-45032633-C-T	c.433G>A;<br>c.1091C>T	p.Glu145Lys;<br>p.Pro364Leu	6.61E-07;<br>6.20E-07	DLBCL and see case report	rituximab (R-CHOP)	Le Voyer et al. 2023 & 2024 (112, 113)
18A	n/a	F	2.4	Cartilage hair hypoplasia (CHH)	157660	250250	< 1	18	19	*RMRP*		NR_003051.4	AR	comp het	9-35657955-G-A<br>9-35657948-T-C	n.63C>T;<br>n.70A>G	n/a	4.71E-05;<br>1.17E-03	AIHA, granulomatosis	corticosteroids, infliximab	Hadjadj et al. 2018 (110)
19A	n/a	M	1.3	SASH3 deficiency	300441	301082	n/a	55	56	*SASH3*		NM_018990.4	XL	hemi	X-129792768-C-T	c.733C>T	p.Arg245Ter	0	aHUS, granulomatosis,<br>leukopenia, thrombocytopenia	corticosteroids, rituximab	Delmonte et al. 2021 (101)
20A	USA	M	6.6	STAT1 GOF	600555	614162	n/a	31	31	*STAT1*		NM_007315.4	AD (GOF)	het	2-190995185-G-C	c.820C>G	p.Arg274Gly	0	dissem. histoplasmosis and see case report	n/a	Sampaio et al. 2013 (121)
20B	USA	F	6.6				2	19	20					het	2-190986876-A-T	c.1199T>A	p.Leu400Gln	0	see case report	n/a	Zerbe et al. 2016 (10)
20C	USA	M	6.6				n/a	20	20					het	2-190986921-G-A	c.1154C>T	p.Thr385Met	0	see case report	rituximab	Zerbe et al. 2016 (10)
20D	USA	M	6.6				n/a	32	32					het	2-190995184-C-T	c.821G>A	p.Arg274Glu	0	see case report	n/a	Zerbe et al. 2016 (10)
21A	Israel	M	7.1	STING-associated vasculopathy, infantile-onset (SAVI)	612374	615934	n/a	43	n/a	*STING1*	*TMEM173*	n/a	AD	het	n/a	n/a	n/a	n/a	RA, interstitial lung disease	corticosteroids, methotrexate, tofacitinib	Schwartzmann et al. 2023 (123)
22A	n/a	M	1.3	STK4 deficiency	604965	614868	1	18	18	*STK4*		NM_006282.5	AR	hom	20-44981932-C-T	c.349C>T	p.Arg117Ter[f]	8.07E-06	eczema, Hodgkin lymphoma	rituximab	Hadjadj et al. 2018 (110)
23A	China	M	3.1	E47 transcription factor deficiency	147141	616941	n/a	28	28	*TCF3*	*E47*	NM_003200.5	AD	het	19-1622069-G-C	c.807C>G	p.His269Gln	1.18E-05	vision loss and see case report	n/a	Li et al. 2022 (114)
24A	UK	M	2.1	Wiskott-Aldrich syndrome (WAS LOF)	300392	301000	12	20	20	*WAS*		NM_000377.3	XL	hemi	n/a	c.155C>T[g]	n/a	n/a	vision loss	methylprednisolone	Downes et al. 2001 (105)
25A	France	M	4.7	XIAP deficiency (XLP2)	300079	300635	65	67	67	*XIAP*		NM_001167.4	XL	hemi	X-123885881-G-A	c.219G>A	p.Trp73Ter	0	MZL and see case report	n/a	Seguier et al. 2021 (38)
26A	Japan	M	2.2	Immunodeficiency with centromeric instability and<br>facial anomalies (ICF type 2)	614064	614069	n/a	39	41	*ZBTB24*		NM_014797.3	AR	hom	6-109476925-G-A	c.958C>T	p.Arg320Ter	7.44E-06	see case report	n/a	Nitta et al. 2013, Kamae et al. 2018 (19, 120)
**Patients diagnosed with hemophagocytic lymphohistiocytosis (HLH) and PML**																					
27A	France	M	n/a	Familial hemophagocytic lymphohistiocytosis	n/a	n/a	n/a	16	alive	unknown	n/a	n/a	n/a	n/a	n/a	n/a	n/a	n/a	FHL, EBV-LPD	cyclosporine, natalizumab	Kumar et al. 2019 (37), FAERS Case ID 10576452
27B	USA	M	n/a	n/a	n/a	n/a	n/a	61	61	unknown	n/a	n/a	n/a	n/a	n/a	n/a	n/a	n/a	HLH (EBV)	corticosteroids, etoposide	Koop et al. 2016 (36)
27C	Japan	M	n/a	n/a	n/a	n/a	n/a	36	alive	unknown	n/a	n/a	n/a	n/a	n/a	n/a	n/a	n/a	HLH, SLE	cyclophosamide, glucocorticoid,<br>mycophenolate mofetil, rituximab	Ishikawa et al. 2018 (35)
**Patients testing positive for a PML risk variant**																					
28A	n/a	F	8	C8 beta deficiency	120960	613789	n/a	n/a	n/a	*C8B*		NM_000066.4	AR	het	1-56943786-C-A	c.1144G>T	p.Asp382Tyr	5.95E-03	MS	natalizumab	Eis et al. 2020, Hatchwell et al. 2022 (5, 6)
28B	n/a	F					n/a	n/a	n/a										HIV	n/a	
28C	n/a	F					n/a	n/a	n/a										CLL	n/a	
28D	n/a	M					n/a	n/a	n/a										HIV	n/a	
28E	n/a	M					n/a	n/a	n/a										HIV	n/a	
28F	n/a	M					n/a	n/a	n/a										MS	natalizumab	
28G	n/a	F					n/a	n/a	n/a										MS	natalizumab	
28H	n/a	F					n/a	n/a	n/a										MS	natalizumab	
28I	n/a	M					n/a	n/a	n/a										HIV	n/a	
29A	n/a	F	n/a	n/a	601624	n/a	n/a	n/a	n/a	*FCN2*		NM_004108.3	n/a	het	9-134887405-G-A	c.932G>A	p.Arg311Gln	4.81E-03	MS	natalizumab	Eis et al. 2020, Hatchwell et al. 2022 (5, 6)
29B	n/a	M					n/a	n/a	n/a										HIV	n/a	
29C	n/a	M					n/a	n/a	n/a										HIV	n/a	
29D	n/a	M					n/a	n/a	n/a										CLL	n/a	
29E	n/a	M					n/a	n/a	n/a										HIV	n/a	
29F	n/a	F					n/a	n/a	n/a										HIV	n/a	
29G	n/a	F					n/a	n/a	n/a										MS	dimethyl fumarate	
29H	n/a	F					n/a	n/a	n/a										B cell lymphoma	rituximab	
29I	n/a	M					n/a	n/a	n/a										Behcet's disease, immune thrombocytopenia	rituximab	
29J	n/a	F					n/a	n/a	n/a										MS	natalizumab	
30A	n/a	F	n/a	n/a	600684	n/a	n/a	n/a	n/a	*LY9*	*CD229, SLAMF3*	NM_002348.4	n/a	het	1-160799805-AG-A	c.182del	p.Gly61ValfsTer3	6.14E-05	HIV	n/a	Eis et al. 2020, Hatchwell et al. 2022 (5, 6)
30B	n/a	M					n/a	n/a	n/a										transplant (liver)	n/a	
30C	n/a	F					n/a	n/a	n/a										MS	natalizumab	
31A	n/a	F	4.1	STXBP2 / Munc18-2 deficiency (FHL5)	601717	613101	n/a	n/a	n/a	*STXBP2*	*MUNC18-2*	NM_006949.4	AR, AD	het	19-7647401-G-C	c.1586G>C	p.Arg529Pro	2.34E-03	MS	natalizumab	Eis et al. 2020, Hatchwell et al. 2022 (5, 6)
31B	n/a	M					n/a	n/a	n/a										Sarcoidosis	n/a	
31C	n/a	F					n/a	n/a	n/a										MS	natalizumab	
31D	n/a	F					n/a	n/a	n/a										HIV, HCV	n/a	

[a] All n/a entries = not available or none

[b] IEI table #s, headings, and sub-headings are based on the 2024 IUIS update: Poli MC et al. J. Hum. Immun. 2025; 1 (1)

Table #	Table heading	Table Sub-heading
1.2	Immunodeficiencies affecting cellular and humoral immunity	T-B-SCID
1.3	Immunodeficiencies affecting cellular and humoral immunity	Combined Immunodeficiency (CID), generally less profound than SCID
2.1	Combined immunodeficiencies with associated or syndromic features	Immunodeficiency with Congenital Thrombocytopenia
2.2	Combined immunodeficiencies with associated or syndromic features	DNA repair defects other than those listed in Table 1
2.4	Combined immunodeficiencies with associated or syndromic features	Immuno-osseous dysplasias
2.7	Combined immunodeficiencies with associated or syndromic features	Anhidrotic Ectodermodysplasia with Immunodeficiency (EDA-ID)
2.9	Combined immunodeficiencies with associated or syndromic features	Other defects
3.1	Predominantly antibody deficiencies	Severe reduction in all serum immunoglobulin isotypes with profoundly decreased or absent B cells, agammaglobulinemia
3.2	Predominantly antibody deficiencies	Severe reduction in at least 2 serum immunoglobulin isotypes with normal or low number of B cells, CVID phenotype
4.3	Diseases of immune dysregulation	Regulatory T Cell Defects
4.7	Diseases of immune dysregulation	Susceptibility to EBV and lymphoproliferative conditions
5.4	Congenital defects of phagocyte number or function	Other Non-Lymphoid Defects
6.6	Defects in intrinsic and innate immunity	Predisposition to mucocutaneous candidiasis
7.1	Autoinflammatory disorders	Type 1 Interferonopathies
7.3	Autoinflammatory disorders	Non-Inflammasome Related Conditions

[c] The allele frequency (AF) of each variant, if known, is reported using the Genome Aggregation Database (gnomAD) population database (Chen S. et al. Nature, 2024 Jan;625(7993):92-100, PMID 38057664). The AF is reported for Total subjects in gnomAD but genetic ancestry subgroups are also available via the gnomAD browser. PML case reports with unreported variants in a given gene are reported with AF = n/a and variants found in PML patients but not found in gnomAD are reported with AF = 0.

[d] Mutation was reported as p.S147fs*6 but this does not match any of the transcript variants for DCLRE1C; possibly this is a mutation of p.M147 (transcript variant NM_001033855.3)

[e] This patient also had a CHD7 mutation

[f] Mutation was reported as p.A117* but is likely R117* as there are no transcript variants with Ala in position 117

[g] Mutation was reported as c.155C>T but this does not match any of the transcript variants (no C in position 155 and this is exon 2 not exon 1 as Downes et al. reported); possibly this is c.154C>T (p.Gln52Ter)
