# Supplementary material for: Genetics of progressive multifocal leukoencephalopathy: update on case reports with an inborn error of immunity and risk variants found in drug-linked cases
Source: Front Neurol. 2025 Jul 15;16:1629581. doi: 10.3389/fneur.2025.1629581 (PMC12320240; doi:10.3389/fneur.2025.1629581)
Supplement: Supplementary file 2 [file Data_Sheet_2.pdf]

SUPPLEMENTARY TABLE 2 | Drug-exposed PML cases reported to the FDA Adverse Event Reporting System (FAERS, 2020-2024) by the original drug manufacturer and drugs with potentially PML-inducing mechanisms (same MOA) that are not currently linked to PML.[a]

Drug name	Drug brand name	Drug approval date FDA	EMA	Drug MOA/target[a]	Indication(s)[b]	Manufacturer/Sender to FAERS	PML in US (FDA) Prescribing Information[c]	PML in European (EMA) Prescribing Information	PML cases in FAERS[d] Total	Died	% Died
**Multiple sclerosis (MS) drugs (or CD and UC)**											
**natalizumab**	**Tysabri**	**23-Nov-04**	**27-Jun-06**	**ITGA4/ITGB1 & ITGA4/ITGB7**	**CD, MS**	**Biogen**	**Boxed Warning**	**Yes**	**231**	**42**	**18%**
fingolimod	Gilenya	21-Sep-10	17-Mar-11	S1P receptor modulator	MS	Novartis	Warnings and Precautions	Yes	51	8	16%
ocrelizumab	Ocrevus	28-Mar-17	8-Jan-18	CD20	MS	Roche	Warnings and Precautions	Yes	28	3	11%
dimethyl fumarate	Tecfidera	27-Mar-13	30-Jan-14	NRF2 activator	MS	Biogen	Warnings and Precautions	Yes	10	1	10%
ofatumumab	Kesimpta	20-Aug-20	26-Mar-21	CD20	MS	Novartis	Warnings and Precautions	Yes	9	0	0%
**rituximab**	**MabThera, Rituxan**	**26-Nov-97**	**2-Jun-98**	**CD20**	**MS (off-label)**	**Roche**	**Boxed Warning**	**Yes**	**7**	**0**	**0%**
siponimod	Mayzent	26-Mar-19	13-Jan-20	S1P receptor modulator	MS	Novartis	Warnings and Precautions	Yes	5	0	0%
diroximel fumarate	Vumerity	29-Oct-19	15-Nov-21	NRF2 activator	MS	Biogen	Warnings and Precautions	Yes	2	0	0%
ozanimod	Zeposia	20-Mar-20	20-May-20	S1P receptor modulator	MS, UC	Bristol Myers Squibb/Celgene	Warnings and Precautions	Yes	2	0	0%
alemtuzumab	Lemtrada	14-Nov-14	12-Sep-13	CD52	MS	Sanofi Aventis	Warnings and Precautions	Yes	1	0	0%
cladribine	Mavenclad	29-Mar-19	22-Aug-17	purine analogue	MS	EMD Serono	Warnings and Precautions	Yes	1	0	0%
ublituximab	Briumvi	28-Dec-22	31-May-23	CD20	MS	TG Therapeutics	Warnings and Precautions	Yes	0	0	n/a
monomethyl fumarate	Bafiertam	30-Apr-20	n/a	NRF2 activator	MS	Banner Life Sciences	Warnings and Precautions	Yes	0	0	n/a
ponesimod	Ponvory	19-Mar-21	19-May-21	S1P receptor modulator	MS	Vanda, Juvise	Warnings and Precautions	Yes	0	0	n/a
**Autoimmune disease drugs (excluding MS)**											
**rituximab**	**MabThera, Rituxan**	**26-Nov-97**	**2-Jun-98**	**CD20**	**CLL, GPA, MPA, NHL, PeV, RA**	**Roche**	**Boxed Warning**	**Yes**	53	18	34%
vedolizumab	Entyvio	20-May-14	22-May-14	ITGA4/ITGB7	CD, UC	Takeda	Warnings and Precautions	Yes	18	1	6%
**efalizumab[e]**	**Raptiva (withdrawn)**	**27-Oct-03**	**20-Sep-04**	**CD11A**	**Ps**	**Genentech/Roche**	**Boxed Warning**	**Yes**	**12**	**5**	**42%**
belimumab	Benlysta	9-Mar-11	13-Jul-11	BLyS inhibitor	lupus nephritis, SLE	GlaxoSmithKline	Warnings and Precautions	Yes	8	0	0%
etanercept	Enbrel	2-Nov-98	2-Feb-00	TNF inhibitor	AS, JIA, nr-AxSpA, Ps, PsA, RA	Amgen	No	No	8	2	25%
azathioprine	Imuran	20-Mar-68	various	purine antagonist	RA, SLE, PeV	Mylan (generic)	Warnings and Precautions	No	6	2	33%
adalimumab	Humira	31-Dec-02	8-Sep-03	TNF inhibitor	AS, CD, HS, JIA, Ps, PsA, RA, UC, UV	AbbVie	No	No	5	1	20%
abatacept	Orencia	23-Dec-05	21-May-07	CD80/CD86	GVHD, JIA, PsA, RA	Bristol Myers Squibb	No	Yes	4	0	0%
eculizumab	Soliris	16-Mar-07	20-Jun-07	complement (C5) inhibitor	aHUS, gMG, NMOSD, PNH	Alexion Pharmaceuticals	No	No	4	4	100%
tofacitinib citrate	Xeljanz	6-Nov-12	22-Mar-17	JAK inhibitor	AS, JIA, PsA, RA, UC	Pfizer	No	No	4	0	0%
upadacitinib	Rinvoq	16-Aug-19	16-Dec-19	JAK inhibitor	AD, AS, PsA, RA, UC	AbbVie	No	No	2	1	50%
inebilizumab	Uplizna	11-Jun-20	2-May-22	CD19	NMOSD	Amgen/Horizon/Viela Bio	Warnings and Precautions	Yes	1	1	100%
leflunomide	Arava	10-Sep-98	2-Sep-99	pyrimidine synthesis inhibitor	RA	Aventis/Sanofi	No	Yes	1	0	0%
etrasimod	Velsipity	13-Oct-23	16-Feb-24	S1P receptor modulator	UC	Pfizer	Warnings and Precautions	Yes	0	0	n/a
voclosporin	Lupkynis	22-Jan-21	15-Sep-22	calcineurin inhibitor	lupus nephritis	Aurinia, Otsuka	No	No	0	0	n/a
avacopan	Tavneos	7-Oct-21	11-Jan-22	complement (C5) inhibitor	GPA, MPA	Amgen	No	No	0	0	n/a
crovalimab	Piasky	24-Jun-24	26-Aug-24	complement (C5) inhibitor	PNH	Roche	No	No	0	0	n/a
pozelimab	Veopoz	18-Aug-23	20-Dec-23	complement (C5) inhibitor	CHAPLE disease	Regeneron	No	No	0	0	n/a
ravulizumab	Ultomiris	21-Dec-18	2-Jul-19	complement (C5) inhibitor	aHUS, NMSOD, PNH	Alexion/AstraZeneca	No	No	0	0	n/a
zilucoplan	Zilbrysq	25-Oct-23	4-Dec-23	complement (C5) inhibitor	gMG	UCB	No	No	0	0	n/a
abrocitinib	Cibinqo	14-Jan-22	9-Dec-21	JAK inhibitor	AD	Pfizer	No	No	0	0	n/a
baricitinib	Olumiant	31-May-18	13-Feb-17	JAK inhibitor	AD, RA	Eli Lilly	No	No	0	0	n/a
filgotinib	Jyseleca	N/A	24-Sep-20	JAK inhibitor	RA, UC	Alfasigma	N/A	No	0	0	n/a
ritlecitinib	Litfulo	23-Jun-23	19-Sep-23	JAK / TK inhibitor (TEC)	GVHD, myelofibrosis, PolV	Pfizer	No	No	0	0	n/a
**Hematological malignancy drugs (mainly)**											
**rituximab**	**MabThera, Rituxan**	**26-Nov-97**	**2-Jun-98**	**CD20**	**CLL, GPA, MPA, NHL, PeV, RA**	**Roche**	**Boxed Warning**	**Yes**	**189**	**74**	**39%**
lenalidomide	Revlimid	27-Dec-05	14-Jun-07	angiogenesis inhibitor	FL, MCL, MM, MZL	Bristol Myers Squibb/Celgene	Postmarketing Experience	Yes	30	14	47%
bendamustine	Treanda, Levact	20-Mar-08	7-Jul-10	alkylating agent	CLL, MM, NHL	Teva	Warnings and Precautions	Yes	28	10	**36%**
daratumumab	Darzalex	16-Nov-15	20-May-16	CD38	MM	Johnson and Johnson	No	No	27	9	33%
**obinutuzumab**	**Gazyva, Gazyvaro**	**13-Nov-13**	**23-Jul-14**	**CD20**	**CLL, FL**	**Roche**	**Boxed Warning**	**Yes**	**27**	**13**	**48%**
**brentuximab vedotin**	**Adcetris**	**19-Aug-11**	**25-Oct-12**	**CD30-MMAE (ADC)**	**ALCL, Hodgkin lymphoma**	**Pfizer/Seattle Genetics**	**Boxed Warning**	**Yes**	**20**	**7**	**35%**
pomalidomide	Pomalyst, Imnovid	8-Feb-13	5-Aug-13	angiogenesis inhibitor	KS, MM	Bristol Myers Squibb/Celgene	Postmarketing Experience	Yes	20	8	40%
venetoclax	Venclexta, Venclyxto	11-Apr-16	5-Dec-16	BCL2 inhibitor	AML, CLL, SLL	AbbVie, Roche	No	No	19	7	37%
axicabtagene ciloleucel	Yescarta	18-Oct-17	23-Aug-18	CD19 (CAR T)	B-cell lymphomas, FL	Gilead	Warnings and Precautions	No	15	8	53%
carfilzomib	Kyprolis	20-Jul-12	19-Nov-15	proteosome inhibitor	MM	Amgen	Warnings and Precautions	Yes	14	7	50%
ruxolitinib	Jakafi, Jakavi	16-Nov-11	23-Aug-12	JAK inhibitor	GVHD, myelofibrosis, PolV	Incyte	Warnings and Precautions	Yes	12	6	50%
bortezomib	Velcade	13-May-03	26-Apr-04	proteosome inhibitor	MCL, MM	Takeda	Postmarketing Experience	Yes	11	4	36%
ibrutinib	Imbruvica	13-Nov-13	21-Oct-14	BTK inhibitor	CLL/SLL, GVHD, MCL, MZL, WM	AbbVie	Warnings and Precautions	Yes	10	1	10%
polatuzumab vedotin	Polivy	10-Jun-19	16-Jan-20	CD79B-MMAE (ADC)	DLBCL	Roche	Warnings and Precautions	Yes	9	4	44%
teclistamab-cqyv	Tecvayli	25-Oct-22	23-Aug-22	BCMA/CD3 (BiTE)	MM	Johnson and Johnson	Clinical Trials Experience	Yes	7	2	29%

Drug name	Drug brand name	Drug approval date FDA	EMA	Drug MOA/target[a]	Indication(s)[b]	Manufacturer/Sender to FAERS	PML in US (FDA) Prescribing Information[c]	PML in European (EMA) Prescribing Information	PML cases in FAERS[d] Total	Died	% Died
thalidomide	Thalomid	16-Jul-98	16-Apr-08	angiogenesis inhibitor	ENL, MM	Bristol Myers Squibb/Celgene	Postmarketing Experience	Yes	7	4	57%
acalabrutinib	Calquence	31-Oct-17	5-Nov-20	BTK inhibitor	CLL/SLL, MCL	AstraZeneca	Warnings and Precautions	Yes	6	2	33%
elotuzumab	Empliciti	30-Nov-15	11-May-16	SLAMF7	MM	Bristol Myers Squibb/Celgene	No	No	5	0	0%
epcoritamab-bysp	Epkinly, Tepkinly	19-May-23	25-Sep-23	CD20/CD3 (BiTE)	DLBCL, FL	Genmab	No	No	4	2	50%
ixazomib	Ninlaro	29-Jan-16	21-Nov-16	proteosome inhibitor	MM	Takeda	No	No	3	2	67%
tisagenlecleucel	Kymriah	30-Aug-17	23-Aug-18	CD19 (CAR T)	ALL, DLBCL	Novartis	No	No	3	2	67%
belantamab mafodotin	Blenrep	5-Aug-20	25-Aug-20	BCMA-MMAF (ADC)	MM	GlaxoSmithKline	No	No	2	1	50%
ciltacabtagene autoleucel	Carvikti	28-Feb-22	25-May-22	BCMA (CAR-T)	MM	Janssen, Legend	No	No	2	1	50%
isatuximab	Sarclisa	31-Mar-21	30-May-20	CD38	MM	Sanofi	No	No	2	0	0%
blinatumomab	Blincyto	3-Dec-14	23-Jan-15	CD19/CD3 (BiTE)	ALL	Amgen	No	Yes	1	1	100%
elranatamb	Elrexfio	14-Aug-23	8-Dec-23	BCMA/CD3 (BiTE)	MM	Pfizer	No	Yes	1	1	100%
fedratinib	Inrebic	16-Aug-19	8-Feb-21	JAK inhibitor	myelofibrosis	Bristol Myers Squibb/Celgene	No	No	1	0	0%
glofitamab-gxbm	Columvi	15-Jun-23	7-Jul-23	CD20/CD3 (BiTE)	DLBCL	Genentech/Roche	No	No	1	0	0%
inotuzumab ozogamicin	Besponsa	17-Aug-17	28-Jun-17	CD22-calicheamicin (ADC)	ALL	Pfizer	No	No	1	1	100%
lisocabtagene maraleucel	Breyanzi	5-Feb-21	4-Apr-22	CD19 (CAR T)	DLBCL, FL, PMBCL	Bristol Myers Squibb	No	No	1	0	0%
tafasitamab-cxix	Monjuvi, Minjuvi	31-Jul-20	26-Aug-21	CD19	DLBCL	Incyte, Novartis/MorphoSys	Clinical Trials Experience	No	1	0	0%
idecabtagene vicleucel	Abcema	26-Mar-21	18-Aug-21	BCMA (CAR-T)	MM	Bristol Myers Squibb	No	No	0	0	n/a
pirtobrutinib	Jaypirca	27-Jan-23	31-Oct-23	BTK inhibitor	MCL	Eli Lilly	No	No	0	0	n/a
zanubrutinib	Brukinsa	31-Aug-21	22-Nov-21	BTK inhibitor	MCL, MZL, WM	Beigene	No	No	0	0	n/a
brexucabtagene autoleucel	Tecartus	24-Jul-20	14-Dec-20	CD19 (CAR-T)	ALL, MCL	Gilead	No	Yes	0	0	n/a
loncastuximab tesirine-lpyl	Zynlonta	23-Apr-21	20-Dec-22	CD19 (ADC)	DLBCL	ADC Therapeutics	No	No	0	0	n/a
obecabtagene autoleucel	Aucatzyl	8-Nov-24	Pending	CD19 (CAR-T)	ALL	Autolus	No	Pending	0	0	n/a
mosunetuzumab-axgb	Lunsumio	22-Dec-22	3-Jun-22	CD20/CD3 (BiTE)	FL	Genentech/Roche	No	No	0	0	n/a
odronextamab	Ordspono	Pending	26-Aug-24	CD20/CD3 (BiTE)	DLBCL, FL	Regeneron	Pending	No	0	0	n/a
ivosidenib	Tibsovo	20-Jul-18	4-May-23	IDH1 inhibitor	AML, CC	Servier/Agios	Clinical Trials Experience	No	0	0	n/a
momelotinib	Ojjara	15-Sep-23	29-Jan-24	JAK inhibitor	myelofibrosis	GlaxoSmithKline	No	No	0	0	n/a
pacritinib	Vonjo	28-Feb-22	N/A	JAK inhibitor	myelofibrosis	Swedish Orphan Biovitrum	No	N/A	0	0	n/a
idelalisib	Zydelig	23-Jul-14	18-Sep-14	PI3K inhibitor (PIK3CD)	CLL	Gilead	No	Yes	0	0	n/a
**Organ transplant drugs (mainly)**											
**mycophenolic mofetil**	**CellCept**	**3-May-95**	**14-Feb-96**	**purine synthesis inhibitor**	**organ rejection prophylaxis**	**Genentech/Roche**	**Boxed Warning**	**Yes**	**27**	**5**	**19%**
cyclosporine	Neoral/Sandimmune	14-Nov-83	various	calcineurin inhibitor	organ rejection prophylaxis, Ps, RA	Novartis	Warnings and Precautions	Yes	11	5	45%
**tacrolimus**	**Prograf, Advagraf**	**8-Apr-94**	**23-Apr-07**	**calcineurin inhibitor**	**organ rejection prophylaxis**	**Astellas**	**Boxed Warning**	**Yes**	**5**	**1**	**20%**
**tacrolimus extend. release**	**Envarsus XR**	**10-Jul-15**	**18-Jul-14**	**calcineurin inhibitor**	**organ rejection prophylaxis**	**Veloxis Pharma.**	**Boxed Warning**	**Yes**	**5**	**2**	**40%**
**belatacept**	**Nulojix**	**15-Jun-11**	**17-Jun-11**	**CD80/CD86**	**organ rejection prophylaxis**	**Bristol Myers Squibb**	**Boxed Warning**	**Yes**	**4**	**2**	**50%**

[a] The Mechanism of Action (MOA) or molecular target for each drug is listed. Abbreviations: ADC = antibody drug-conjugate, BCL2 = BCL2 apoptosis regulator, BCMA = B cell maturation antigen, BiTE = bispecific T cell engager, BLyS = B lymphocyte stimulator (gene symbol is *TNFSF13B*), BTK = Bruton tyrosine kinase, CAR T = chimeric antigen receptor T cell, CD20 (gene symbol now MS4A1, membrane spanning 4-domains A1), CTLA4 = cytotoxic T-lymphocyte associated protein 4, G-CSF = granulocyte colony stimulating factor (gene symbol now *CSF3, colony stimulating factor 3*), HER2 = human epidermal growth factor receptor 2 (gene symbol now ERBB2, erb-b2 receptor tyrosine kinase 2), ITGA4 = integrin subunit alpha 4 (natalizumab binds ITGA4 of heterodimers ITGA4-ITGB1 and ITGA4-ITGB7, vedolizumab binds heterodimer ITGA4-ITGB7), JAK = Janus kinase, K+ = potassium, MMAE = monomethyl auristatin E, MMAF = monomethyl auristatin F, MTOR = mechanistic target of rapamycin, NRF2 = nuclear factor (erythroid-derived 2)-like 2 (gene symbol now NFE2L2, NFE2 like bZIP transcription factor 2), PD-1 = programmed cell death 1 (gene symbol now PDCD1), PD-L1 = programmed cell death 1 ligand 1 (gene symbol now CD274), PI3K = phosphoinositide 3-kinase, RANKL = receptor activator of nuclear factor kappa-B ligand (gene symbol now TNFSF11, TNF superfamily member 11), S1P = sphingosine 1-phospate, TK = tyrosine kinase, TNF = tumor necrosis factor.

[b] Drug indication(s) listed in the prescribing information (aka package insert), abbreviations: aHUS = atypical hemolytic uremic syndrome, ALCL = Anaplastic Large Cell Lymphoma, ALL = Acute Lymphoblastic Leukemia, AML = Acute Myeloid Leukemia, AS = Ankylosing Spondylitis, CD = Crohn's Disease, CLL = Chronic Lymphocytic Leukemia, CLL/SLL = Chronic Lymphocytic Leukemia/Small Lymphocytic Lymphoma, CML = Chronic Myeloid Leukemia, CRS = Cytokine Release Syndrome, DLBCL = Diffuse Large B-Cell Lymphoma, ENL = erythema nodosum leprosum, ES-SCLC = Extensive-Stage Small Cell Lung Cancer, FL = Follicular Lymphoma, GCA = Giant Cell Arteritis, gMG = generalized Myasthenia Gravis, GPA = Granulomatosis with Polyangiitis, GVHD = Graft Versus Host Disease, HS = Hidradenitis Suppurativa, JIA = Juvenile Idiopathic Arthritis, KS = AIDS-related Kaposi Sarcoma, MCL = Mantle Cell Lymphoma, MDS = Myelodysplastic Syndrome, MM = Multiple Myeloma, MPA = Microscopic Polyangiitis, MS = Multiple Sclerosis, MZL = Marginal Zone Lymphoma, NHL = Non-Hodgkin's Lymphoma, NMOSD = Neuromyelitis Optica Spectrum Disorder, nr-AxSpA = Non-Radiographic Axial Spondyloarthritis, NSCLC = non-small cell lung cancer, PAH = pulmonary arterial hypertension, PMBCL = Primary Mediastinal large B-cell Lymphoma, PNH = Paroxysmal Nocturnal Hemoglobinuria, PolV = polycythemia vera, Ps = Plaque Psoriasis, PsA = Psoriatic Arthritis, PV = Pemphigus Vulgaris, RA = Rheumatoid Arthritis, SLE = Systemic Lupus Erythematosus, SLL = Small Lymphocytic Lymphoma, SSc-ILD = Systemic Sclerosis-Associated Interstitial Lung Disease, UC = Ulcerative Colitis, UV = Uveitis, WM = Waldenström's Macroglobulinemia.

[c] Location in the drug prescribing information (aka package insert) of the warning for progressive multifocal leukoencephalopathy (PML); a Boxed Warning (aka Black Box Warning, yellow-highlighted entries) is the strongest FDA drug label warning.

[d] FAERS data are reported for the last 5 years (2020-2024).

[e] Efalizumab (Raptiva) is pink-highlighted because it was withdrawn by regulators in Canada and the EU in Feb-2009 and it was voluntarily withdrawn from the US market by Roche in Apr-2009 due to a high frequency of PML cases.
