# Supplementary material for: Genetics of progressive multifocal leukoencephalopathy: update on case reports with an inborn error of immunity and risk variants found in drug-linked cases
Source: Front Neurol. 2025 Jul 15;16:1629581. doi: 10.3389/fneur.2025.1629581 (PMC12320240; doi:10.3389/fneur.2025.1629581)
Supplement: Supplementary file 3 [file Data_Sheet_3.pdf]

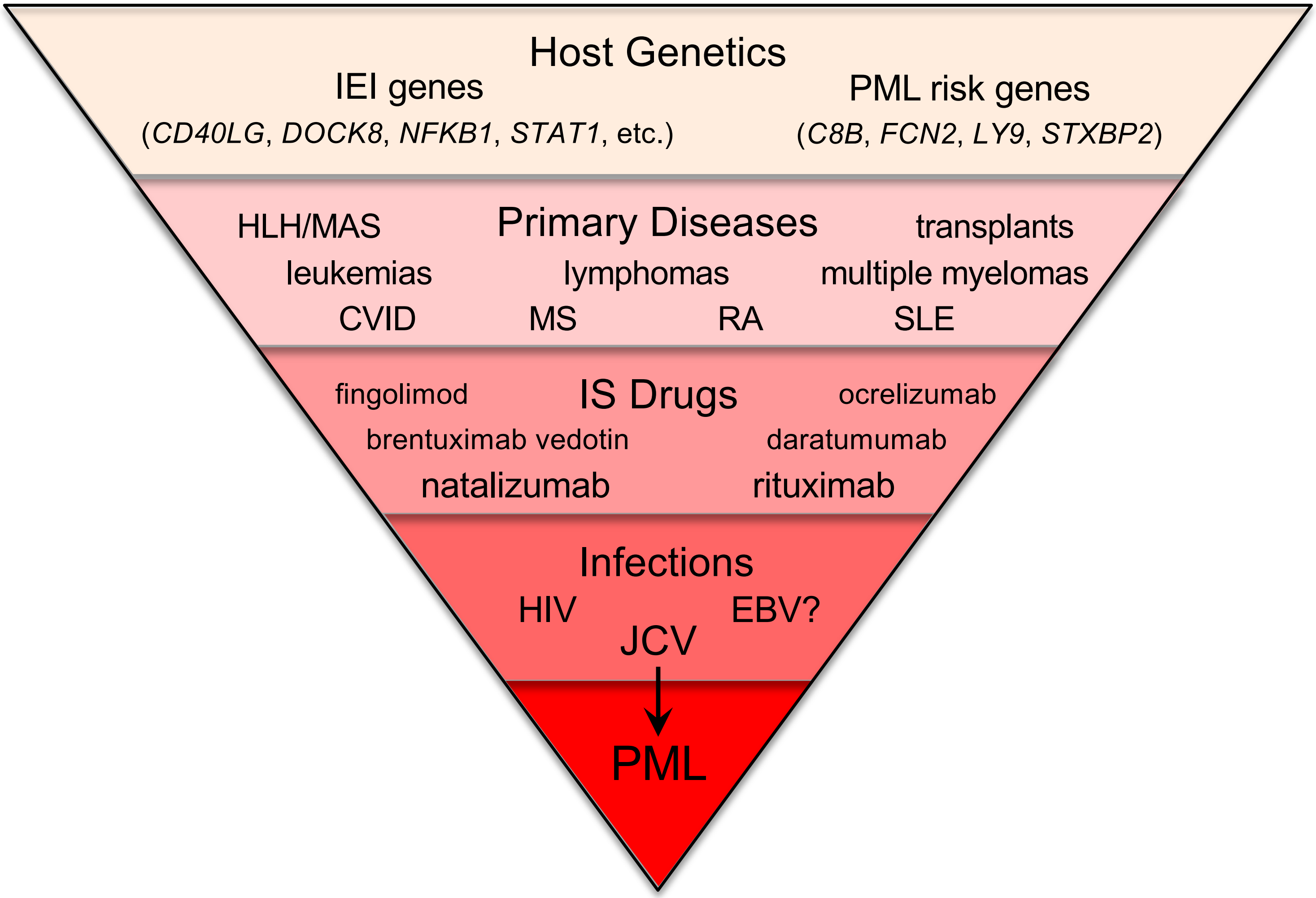

SUPPLEMENTARY FIGURE 1

Constellation of progressive multifocal leukoencephalopathy (PML) risk factors. Abbreviated summary of 4 key factors that increase the risk of PML (not all genes, diseases, drugs, and infections are shown): 1) host genetics (see Figures 1 and 2, and Supplementary Table 1); 2) primary diseases (CVID = common variable immunodeficiency, HLH/MAS = hemophagocytic lymphohistiocytosis/macrophage activation syndrome, MS = multiple sclerosis, RA = rheumatoid arthritis, SLE = systemic lupus erythematosus), 3) immunosuppressant (IS) drugs (see Supplementary Table 2), and 4) infections, JCV is the cause of PML but HIV greatly increases the risk and EBV potentially increases the risk but more studies are needed.
